# The Mediator Subunit OsMED16 Interacts with the WRKY Transcription Factor OsWRKY45 to Enhance Rice Resistance Against *Magnaporthe oryzae*

**DOI:** 10.1186/s12284-024-00698-9

**Published:** 2024-04-01

**Authors:** Yanfei Wu, Yuquan Fu, Zhonglin Zhu, Qin Hu, Feng Sheng, Xuezhu Du

**Affiliations:** 1 State Key Laboratory of Biocatalysis and Enzyme Engineering, School of Life Sciences, Hubei University, Wuhan, 430062 China; 2 Hubei Hongshan Laboratory, Wuhan, 430070 China; 3 State Key Laboratory for Conservation and Utilization of Subtropical Agri-Biological Resources, Guangxi Key Lab for Sugarcane Biology, College of Agriculture, Guangxi University, Nanning, China

**Keywords:** Rice, *OsMED16*, *Magnaporthe oryzae*, Phytoalexin synthesis genes

## Abstract

Rice blast, caused by *Magnaporthe oryzae* (*M. oryzae*), is one of the most common and damaging diseases of rice that limits rice yield and quality. The mediator complex plays a vital role in promoting transcription by bridging specific transcription factors and RNA polymerase II. Here, we show that the rice mediator subunit OsMED16 is essential for full induction of the diterpenoid phytoalexin biosynthesis genes and resistance to the ascomycetous fungus *M. oryzae*. Mutants of *Osmed16* show reduced expression of the DP biosynthesis genes and are markedly more susceptible to *M. oryzae*, while transgenic plants overexpressing *OsMED16* increased the expression of the DP biosynthesis genes and significantly enhanced resistance to *M. oryzae*. Interestingly, OsMED16 is physically associated with the WRKY family transcription factor OsWRKY45, which interacts with the phytoalexin synthesis key regulator transcription factor OsWRKY62. Further, OsMED16-OsWRKY45-OsWRKY62 complex could bind to the promoter regions of phytoalexin synthesis-related genes and activate their gene expression. Our results show that *OsMED16* may enhance rice tolerance to *M. oryzae* via directly manipulating phytoalexin de novo biosynthesis.

**Supplementary Information:**

The online version contains supplementary material available at 10.1186/s12284-024-00698-9.

## Background

Rice blast, caused by the fungal pathogen *M. oryzae*, is considered one of the most destructive diseases of cultivated rice and causes an average annual loss of rice yield of up to 20% (Saleh et al. 2014). The cultivation of resistant varieties is the most practical and cost-effective way to protect against rice blast. However, due to the diversity and rapid variation of *M. oryzae*, the resistance of rice varieties often decreases or loses after several years of application. Therefore, the continuous exploration of rice blast resistance genes will provide theoretical guidance and germplasm resources for rice blast resistance breeding, which is essential for safe rice production.

Phytoalexin are low-molecular-weight pathogen-resistant compounds that are synthesized endogenously in plants after pathogen infection (Paxton 1980). Diterpenoid phytoalexin (DP) has been discovered in a variety of dicots, including elm trees (*Ulmus americana*), cotton (Gossypium hirsutum), tobacco (*Nicotiana tabacum*), sweet potatoes (*Ipomoea batatas*) (Schmelz et al. 2014) and so on. Diterpenoids members currently identified in rice include oryzalexins A–F (Tadami et al. 1985; Sekido et al. 1986; Hideki et al. 1993; Kato et al. 1994), momilactones A and B (Cartwright et al. 1981), phytocassanes A–E (KOGA et al. 1995, 1997), oryzalexin S (Kodama et al. 1992) and ent-10-oxodepressin (Inoue et al. 2013). Phytoalexins are considered to have a crucial role in plant defense mechanisms and serve as molecular markers of plant resistance to disease. Several plants produced a variety of phytoalexins for responding to pathogenic infections (Ahuja et al. 2012). In rice, the antifungal activity against *M. oryzae* is a very significant biological activity of phytoalexins. The stockpile of phytoalexins has occurred quickly in rice after infestation by the *M. oryzae* (Ahuja et al. 2012; Schmelz et al. 2014). Moreover, exogenous momilactones A and B and ent-10-oxodepressin were found to have an inhibitory effect on the growth of *M. oryzae* (Cartwright et al. 1977; Inoue et al. 2013).

The synthesis-related genes of diterpenoid phytoalexin have been extensively reported (Yamane et al. 2013). Cytochromes P450 monooxygenases (*CYP99A2*, *CYP99A3*), copalyl diphosphate synthase (*CPS3*, *CPS4*), momilactone A synthase (*MAS*, *MAS1*), and kaurene synthase-like (*KSL2*, *KSL3*, *KSL7*, *KSL10*) (Shimono et al. 2007), have been shown to be involved in the momilactones biosynthesis. Moreover, the ent-kaurene oxidase paralogs (*KOL1*, *KOL4*, *KOL5*) were reported to contribute to participated in the oryzalexins A–C and E and phytocassanes A–E biosynthesis (Wang et al. 2012). *CYP71Z7*, *CYP76M7* and *CYP76M8* (Cho et al. 2004; Swaminathan et al. 2009; Wu et al. 2011) were shown to participate in the phytocassanes A–E biosynthesis.

In rice, the expression of DP biosynthesis genes were regulated in a *OsWRKY45*-dependent manner. More diterpenoid phytoalexin were synthesized in *OsWRKY45* overexpression plants after *M. oryzae* infection (Akagi et al. 2014). The transcription factor of a basic helix-loop-helix (bHLH)-type named the diterpenoid phytoalexin factor (DPF) has been identified in rice, which is induced by *M. oryzae* and regulates the expression of diterpenoid phytoalexin biosynthetic genes (Yamamura et al. 2015). *OsWRKY62* is a group IIa WRKY transcription factor, and the transcription of *OsWRKY62* is regulated directly or indirectly by *OsWRKY45*. *OsWRKY62* and *OsWRKY45* transcriptions are simultaneously upregulated during benzothiadiazole (BTH) treatment and *M. oryzae* infection (Nakayama et al. 2013). *OsWRKY62* acts as a positive defense regulator to activate the transcription of *OsDPF* genes (a transcription factor regulating diterpenoid phytoalexin biosynthetic genes) when it forms a heterotrimeric complex with the OsWRKY45 (Fukushima et al. 2016). However, whether and how OsWRKY45 cooperates with the mediator complex to activate diterpenoid phytoalexin synthesis genes remains unclear.

When plants are infected with pathogens, the balance of plant development and immunity is disrupted, and the organism will undergo a series of transcriptional reprogramming to activate defense-related gene expression (Katagiri 2004), The mediator complex, a conserved functional multiple protein complex, plays an important role to recruit RNA Pol II and particular transcription factors. The mediator has been widely studied in yeast and multicellular animals, but relatively delayed in plants (Bckstrm et al. 2007; Mathur et al. 2011; Ren et al. 2020; Hu et al. 2021a, b). Some mediator subunits have been found to be involved in immune resistance to various plant pathogens. *AtMED25* act as a positive regulator in JA/ET signaling by interacting with MYC2 and COI1 in Arabidopsis to activate plant resistance against *A. brassicicola and B. cinerea* (Kidd et al. 2009; Cevik et al. 2012). *AtMED14* positively regulates SA responsiveness and *AtMED14* mutants show suppressed *PR1* expression and reduced immune resistance to the *Pseudomonas syringae* (Canet et al. 2012; Zhang et al. 2013).

*MED16*, one of the tail subunits of the mediator, is widely involved in a variety of biological regulation, including flowering time, organ growth and development, and defense against a variety of biotic and abiotic stresses (Zhang et al. 2012; Wang et al. 2016; Dolan et al. 2017). The Arabidopsis *MED16* both controls SA- and JA-mediated defense gene expression and plant immunity to *Pseudomonas syringae* (Zhang et al. 2012). It has been found that the overexpression of *OsSFR6* (*OsMED16* 3513 bp) in the *atsfr6* mutant background restored the visible phenotype of smaller plants of the wild type and restored the expression level of the wild-type COR gene and tolerance to osmotic stress and freezing stress (Wathugala et al. 2011). In rice, Zhang et al. found that VIGS silenced *OsMED16* plants were more susceptible to rice blast disease (Zhang et al. 2021). Overexpression of *OsMED16* (3906 bp) resulted in growth inhibition, yield reduction and the appearance of lesion mimic phenotype. RNA-Seq analysis showed that overexpression of *OsMED16* resulted in the up-regulation of a large number of disease resistance-related genes (Jiang et al. 2021). However, recently a study has shown that overexpression of *OsMED16* (3906) in japonica rice Zhonghua 11 “ZH11” did not exhibit lesion mimic phenotype and showed more susceptibility to rice blast (Zhang et al. 2023). In our study, using overexpression and gene knockout methods, we found that *OsMED16* plays an important role in the expression of DP biosynthesis genes and rice blast tolerance. Further biochemical analyzes were performed to explore how OsMED16 cooperates with OsWRKY45-OsWRKY62 to synthetically activate DP biosynthesis genes Transcription.

## Results

### *OsMED16 *Positively Regulates Rice Resistance Against *M. oryzae*

Because the MED is widely associated with plant immune regulation, the tail subunits play an essential role in the recruitment of specific transcription factors to the target promoter regions (Zhang et al. 2012; Liu et al. 2019). Further, we cloned the full-length CDS of the *OsMED16* (or named *OsSFR6*, which was reported by Wathugala et al. (2011)) from Wild-Type *Nipponbare* rice, which consists of 3513 nucleotides and encodes a protein of 1170 amino acids, 393 bp shorter than the version in the Rice Genome Annotation Project database. The phylogenetic analysis of the sequence of the protein revealed that the homologous sequence of OsMED16 was closest to BdMED16 (*Brachypodium distachyon*), followed by the MED16 protein from maize (Additional file 1: Fig. S2).

**Fig. 1:**
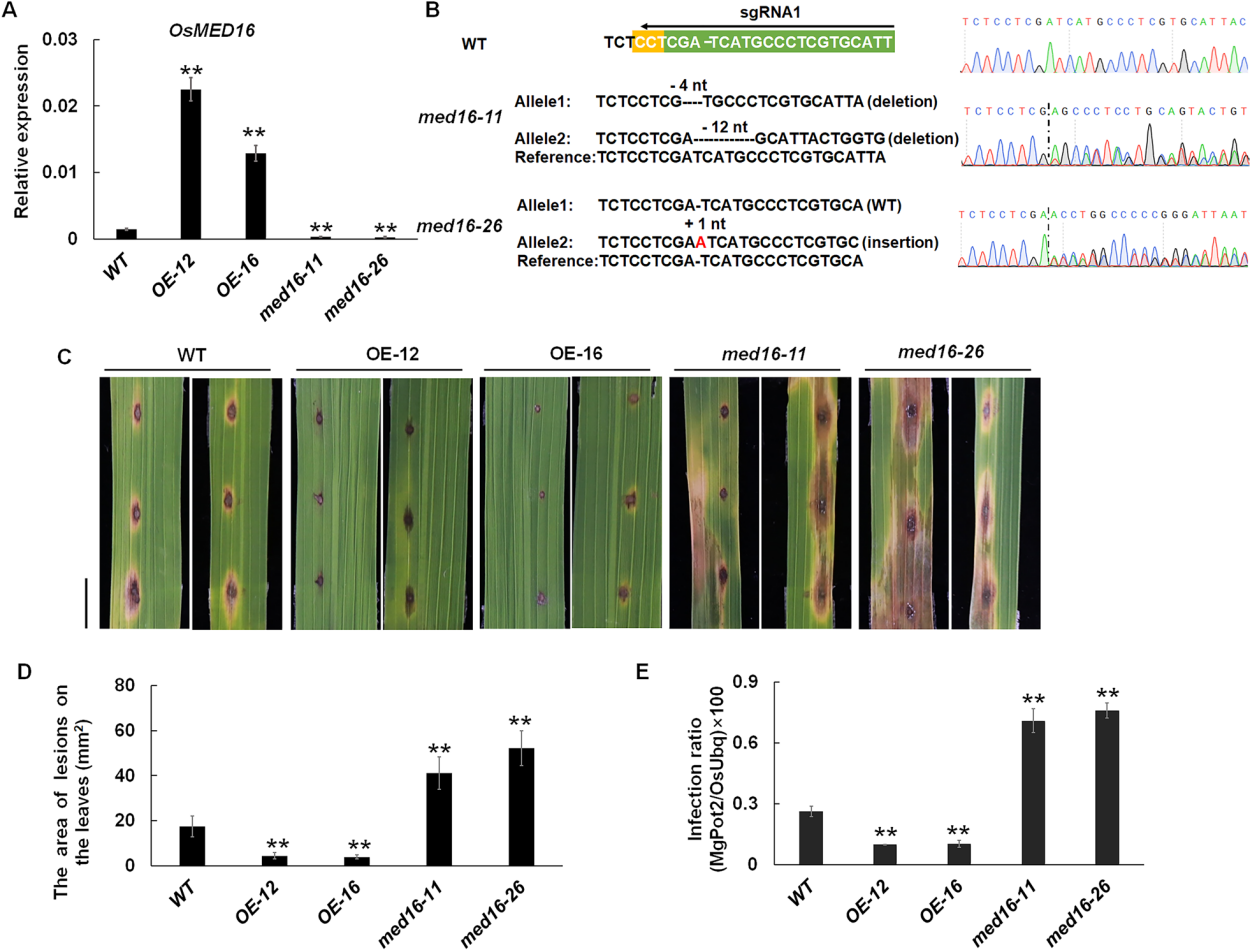
*OsMED16* positively regulates rice resistance against *M. oryzae.* **A** Expression of OsMED16 in WT, OE-12,OE-16 and *med16-11*, *med16-26* plants was analyzed by RT-qPCR. Values were taken as mean ± SD, n = 3, and *OsActin* was used as a reference gene for normalization. **B** The results of Sanger sequencing indicated that the single strand of the sgRNA1 region in *med16-11* and *med16-26* genomic DNA contained deletions of 4 nt and insertions of 1 nt, respectively, compared to the wild type. **C** *OsMED16* overexpression (OE-12, OE-16) enhanced resistance to *M. oryzae*, and CRISPR-Cas9 plants (*med16-11, med16-26*) reduced resistance to *M. oryzae*. WT, OE-12, OE-16 and *med16-11*, *med16-26* plants were inoculated with *M. oryzae* Guy11, and images were taken at 5 dpi. bar = 1 cm. **D** Lesion area of leaves in WT, OE-12, OE-16, and *med16-11*, *med16-26* after inoculation. The values are mean ± SD (n = 15); **p value < 0.01, Student's t-test. **E** Quantitative detection of rice blast fungus growth in WT,OsMED16 overexpression and knockout plants 7 days after infection with strain Guy11. The values are mean ± SD (n = 15); **p value < 0.01, Student's t-test

To verify whether *OsMED16* modulates rice immunity to pathogens, two independent transgenic *OsMED16* constitutively over-expression lines (OE-12 and OE-16) and two independent CRISPR/Cas9 knock-out lines (*med16-11* and *med16-26*) were inoculated with *M. oryzae* isolate *Guy11* in the laboratory. As previously reported (Jiang et al. 2021), the CRISPR/Cas9-edited *OsMED16* orthologous plants grew extremely weakly at the seedling stage and failed to complete the fertility cycle (Additional file 1: Fig. S1). Interestingly, we found that lethality did not occur when one strand of the *Med16* gene was effectively edited (*med16-11* and *med16-26*, Fig. 1B), while RT-qPCR assays showed that the expression level of *OsMED16* in *med16*-*11* and *med16*-*26* was lower than that of the wild type (Fig. 1A). The results of *Guy11* inoculation indicated that overexpression of *OsMED16* strengthened rice resistance to *Guy11*, and the knockout lines exhibited weaker resistance to the disease (Fig. 1C–E), showing that *OsMED16* has a positive regulatory effect on blast resistance in rice. It was recently reported that over-expression of the *OsMed16* gene (3906 bp) results in spontaneous cell death in the leaf blade and sheath with a constitutionally activated immune response (Jiang et al. 2021). Although the overexpression plants of *OsMED16* (3513 bp) also showed enhanced resistance to *M. oryzae*, there was no spontaneous cell death in the leaves, which may result from the truncated 131 amino acids residues at the N-terminal of OsMED16.

### OsMED16 Interacts with OsWRKY45 in the Nucleus

**Fig. 2:**
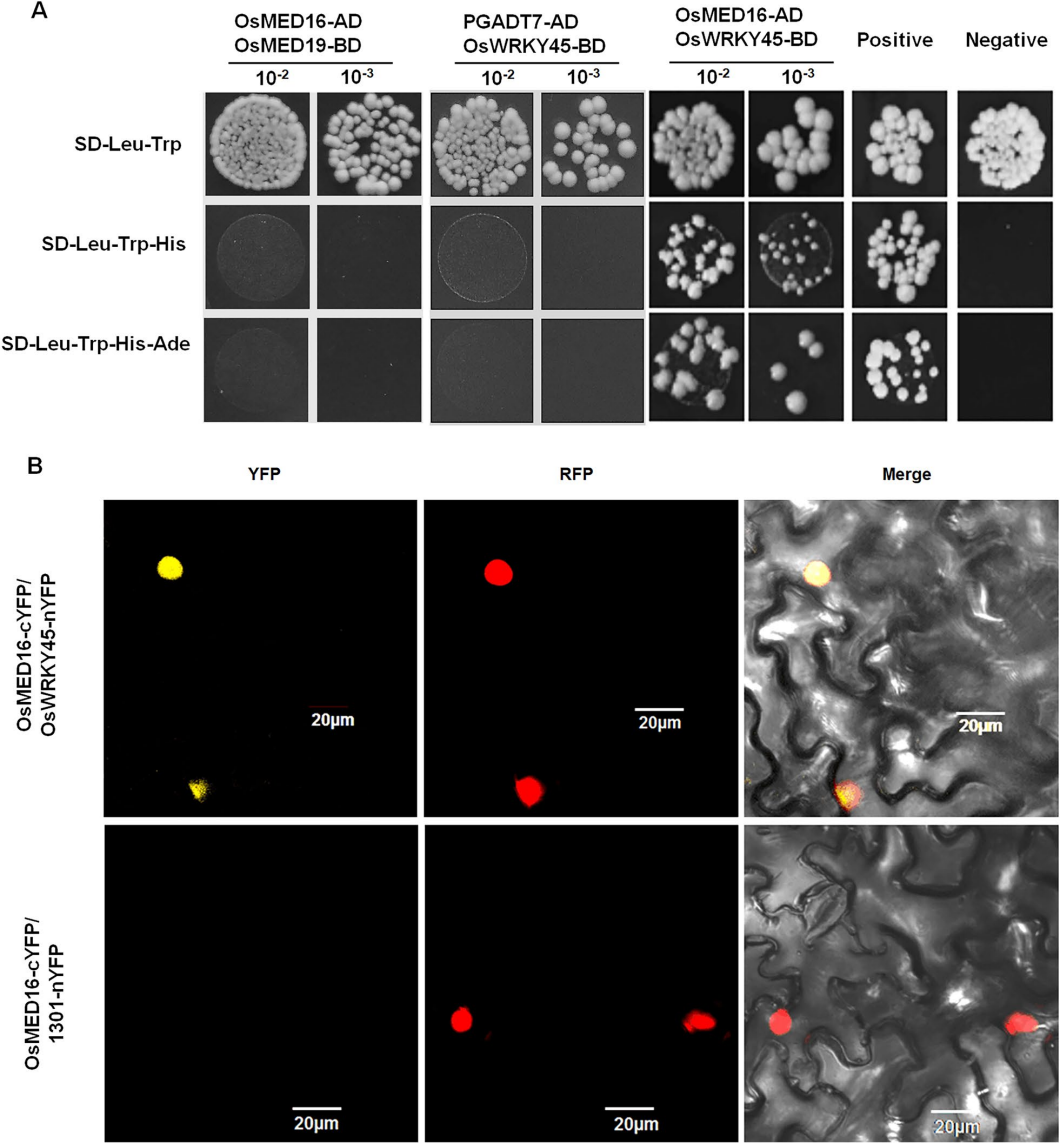
OsMED16 interacts with OsWRKY45 in the nucleus. **A** Y2H assays showed that OsMED16 interacted with OsWRKY45. PGADT7 did not interact with OsWRKY45-BD indicating that OsWRKY45-BD cannot auto-activate the reporter in yeast (*Saccharomyces cerevisiae*). Transformed yeast cells were grown on synthetic glucose (SD) medium, and growing colonies on SD-Trp-Leu-His and SD-Trp-Leu-His-Ade indicate positive interactions 10^–2^, diluted 100 times. 10^–3^, diluted 1000 times. p53-BD/SV40-T antigen-AD was used as positive control, Lam-BD/SV40-T antigen-AD was used as negative control. **B** BIFC analysis showing that the interaction between OsMED16-cYFP and OsWRKY45-nYFP forms a functional YFP in the nucleus. RFP: nuclear localization Marker RFP fluorescent signal; Merge: fused YFP with RFP fluorescent signal. All assays have been repeated at least three times with similar results. bar = 20 μm

For further investigation of the possible mechanisms by which OsMED16 regulates disease resistance in rice, full-length OsMED16 was used to screen a yeast two-hybrid (Y2H) library of rice to identify potential interacting proteins. Among those candidates, OsWRKY45 (Os05t0322900-01) was found to interact with OsMED16. And the 1:1 yeast two hybrid was performed between OsMED16 and OsWRKY45 to confirm this interaction (Fig. 2A). The interaction between OsMED16 and OsWRKY45 in vivo was confirmed by biomolecular fluorescence complementation (BiFC) assays, and the complex occurred in the nucleus (Fig. 2B).

### OsMED16 and OsWRKY45 Synergistically Activate Diterpenoid Phytoalexin Biosynthesis Genes Transcription

**Fig. 3:**
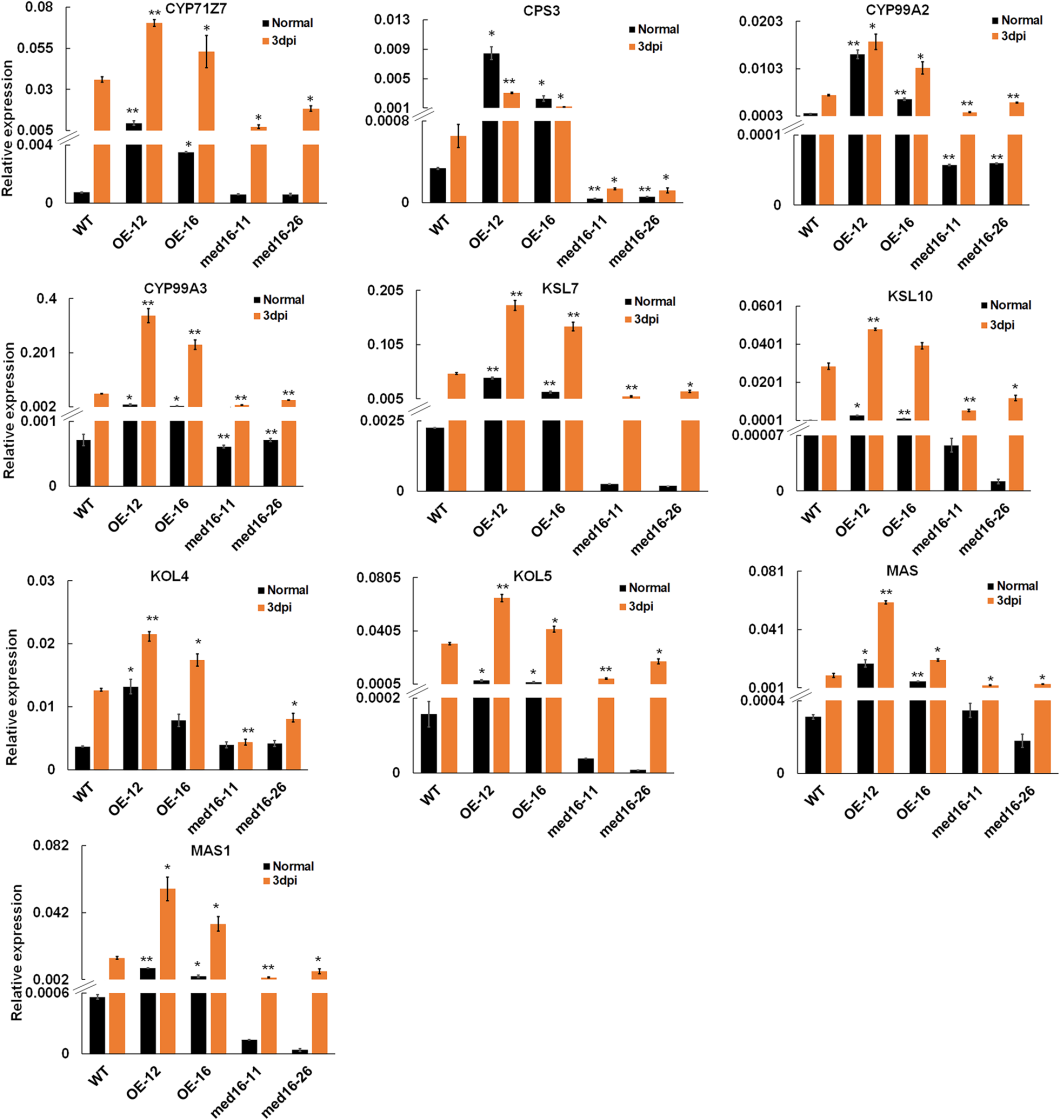
*OsMED16* positively regulates the expression of diterpenoid phytoalexin biosynthesis genes. RT-qPCR analysis of the expression levels of diterpenoid phytoalexin synthesis-related genes in transgenic and wild-type plants under normal and *M. oryzae* infection conditions. The values are the mean ± SD (n = 3); *p value < 0.05, **p value < 0.01, student’s t-test

The expression levels of the genes involved in rice diterpenoid phytoalexin (DP) synthesis in rice were detected in the leaves of wild-type and transgenic plants using RT-qPCR. The results revealed that diterpenoid phytoalexin biosynthesis genes were up-regulated in *OsMED16* overexpressing plants and down-regulated in *OsMED16* knockout plants under normal and *M. oryzae* infection conditions (Fig. 3).

**Fig. 4:**
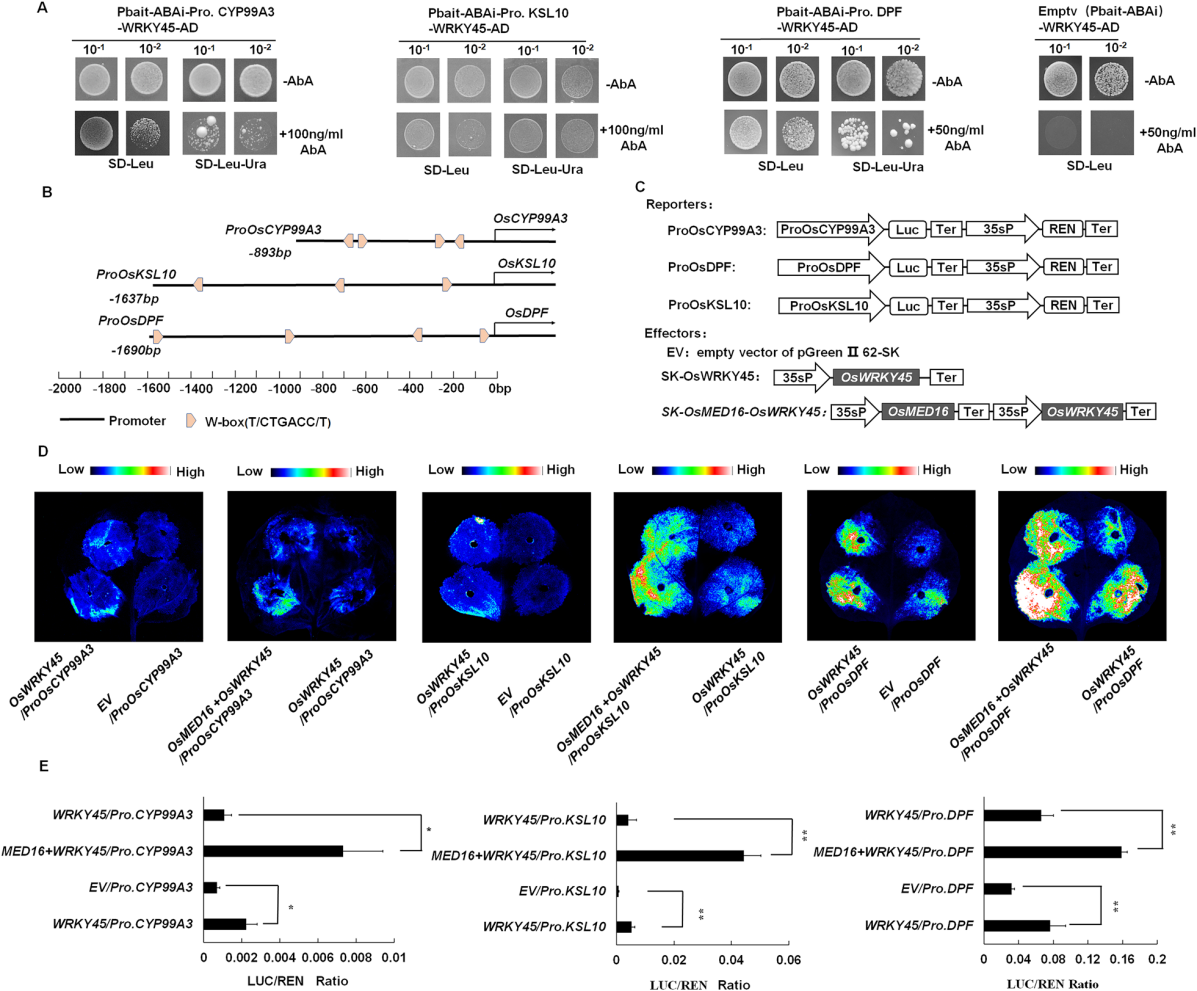
OsMED16 and OsWRKY45 synergistically activate diterpenoid phytoalexin biosynthesis genes transcription. **A** Y1H experiment indicating binding of OsWRKY45 to the promoters of *OsCYP99A3*, *OsKSL10* and *OsDPF*. Negative control was performed with the empty vector pbait-ABA. Yeast cells were grown on SD-Leu and SD-Leu-Ura containing different concentrations of Aureobasidin A (AbA) at 30 °C for 3–5 d. 10^–1^, diluted 10 × 10^–2^, diluted 100 times. **B** W box position diagram of *OsCYP99A3, OsKSL10* and *OsDPF* promoters. **C** Schematic diagram of the trans-activation assay effector and reporter constructs. The reporter vector carries the renilla luciferase gene under the control of the 35S promoter and the firefly luciferase reporter gene under the control of the *OsCYP99A3, OsKSL10*, and *OsDPF* promoters. 35sP, cauliflower mosaic virus 35S promoter; REN, Renilla luciferase; Ter, cauliflower mosaic virus terminator; LUC, firefly luciferase. **D** Analysis of the effects of OsWRKY45 and OsMED16 on *proOsCYP99A3*, *proOsKSL10* and *proOsDPF*. Luminescence imaging was performed in leaves of *N. benthamiana* at 80 h after co-infiltration with the indicated vectors. **E** Luciferase activity analysis using 35S: OsWRKY45 and 35S: OsMED16-OsWRKY45 as effectors and *proOsCYP99A3:Luc*, *proOsKSL10:Luc*, and *proOsDPF: Luc* as reporters, respectively. Values of LUC/REN activity ratio are mean ± SD; n = 3. The Student's t-test was used for comparison. *P < 0.05; **P < 0.01

Previous research has indicated that *OsWRKY45* can regulate diterpenoid phytoalexin synthesis genes expression, and improve rice resistance to rice blast (Akagi et al. 2014). Thus, we further investigated the role of the OsMED16-OsWRKY45 complex in regulating the transcription of genes involved in DP synthesis. According to the DP biosynthesis-related gene expression profiles in the *OsMED16* transgenic lines, we cloned the promoters of several DP biosynthesis-related genes (approximately 2000 bp in the upstream of ATG) including *proOsCSP2*, *proOsCSP4*, *proOsCYP71Z6*, *proOsCYP71Z7*, *proOsCYP99A2*, *proOsCYP999A3*, *proOsKSL4*, *proOsKSL7*, *proOsKSL10*, *proOsMAS1*, and *proOsDPF* which was reported as a basic helix–loop–helix (bHLH)-type transcription factor involved in directly regulating diterpenoid phytoalexin genes (Yamamura et al. 2015). As shown in Additional file 1: Figs. S5 and Fig. 4A, Y1H assays show that OsWRKY45 was able to bind directly to *proOsCYP99A3* (893 bp upstream of ATG), *proOsKSL10* (1637 bp upstream of ATG) and *proOsDPF* (1690 bp in the upstream of ATG) Sequence analysis indicated that there was at least one W-box in the promoter region of *OsCYP99A3*, *OsKSL10*, and *OsDPF* (Fig. 4B). As a control, OsMED16 could not interact with *proOsCYP99A3*, *proOsKSL10* and *proOsDPF* (Additional file 1: Fig. S11). Further, The firefly luciferase (LUC) driven by the DP biosynthesis-related genes promoters (*proOsCYP99A3::LUC*, *proOsKSL10::LUC*, and *proDPF::LUC*) and renilla luciferase (REN) driven by 35S promoter (35S: REN) were transformed transiently into *N.benthamiana* leaves with 35S: OsWRKY45 or empty vector (pGreen II 62-SK), respectively (Fig. 4C and Additional file 1: Fig. S7). The promoter activity was expressed as Luc luminescence intensity and the ratio of Luc activity to Renilla luciferase (REN) activity. The result shows that compared with the control vector, the expression levels of *proOsCYP99A3::LUC*, *proOsKSL10::LUC* and *proDPF::LUC* were significantly activated in the presence of OsWRKY45 (Fig. 4D, E). Our results suggest that 35S: OsWRKY45 could indeed bind to the promoters of *OsCYP99A3, OsKSL10* and *OsDPF* to activate the transcription of a downstream reporter gene. Moreover, in the presence of OsMED16, The luminescence intensity of firefly luciferase of *proOsCYP99A3::LUC*, *proOsKSL10::LUC* and *proOsDPF::LUC* was further enhanced (Fig. 4D, E), indicating that OsMED16 could recruit more RNA polymerase II and enhance the regulatory effect of OsWRKY45 on the promoter. In conclusion, our results show that co-expression of OsMED16 and OsWRKY45 could elevate the DP biosynthesis-related gene promoters’ transcriptional activity.

### OsMED16-OsWRKY45-OsWRKY62 Complex can Further Synergistically Activate Diterpenoid Phytoalexin Biosynthesis Genes Transcription

**Fig. 5:**
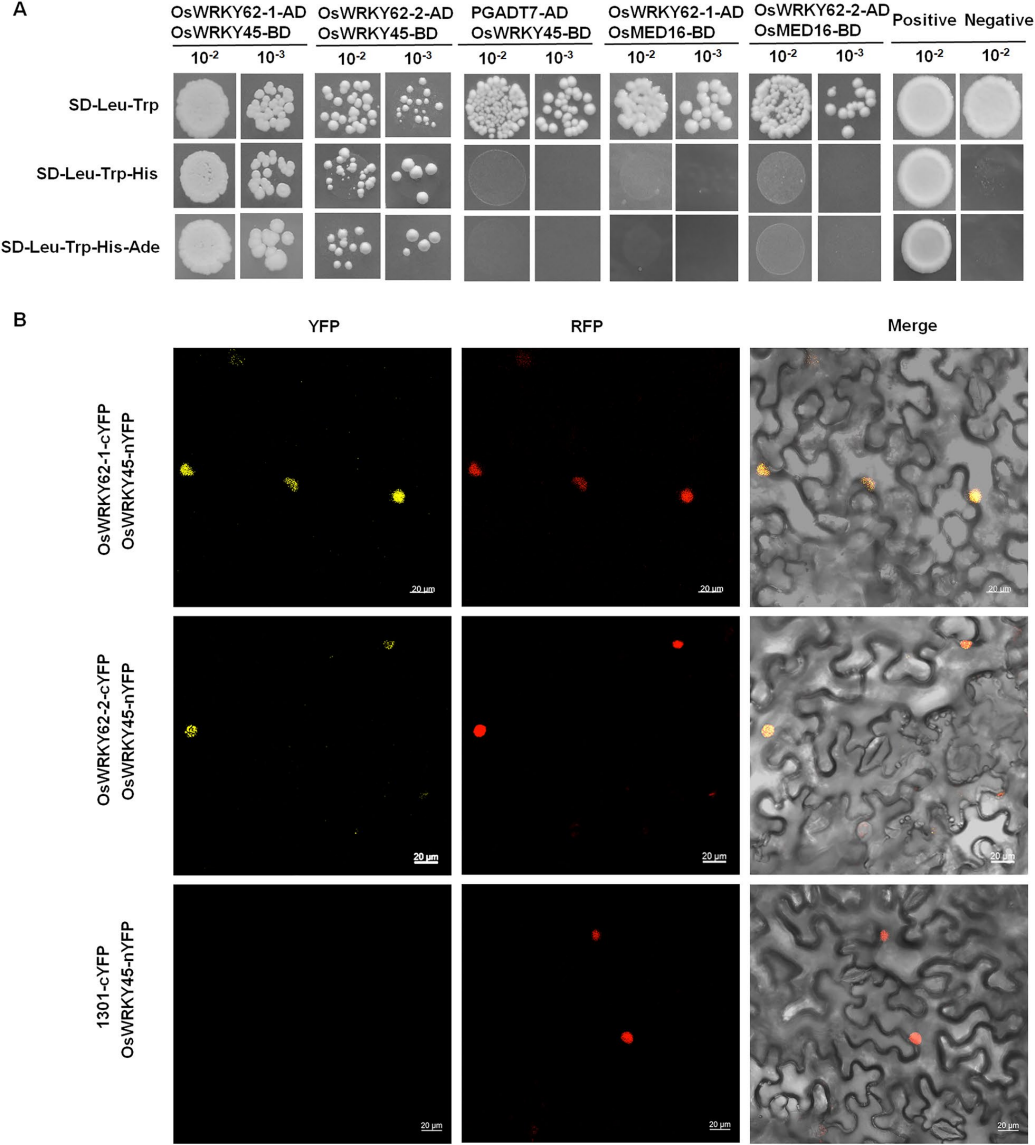
OsWRKY62 interacts with OsWRKY45, but not with OsMED16. **A** Y2H assays showed that OsWRKY62-1 and OsWRKY62-2 interacted with OsWRKY45, OsWRKY62-1 and OsWRKY62-2 did not interact with OsMED16. Positive interaction between colonies of transformed yeast cells grown on synthetic glucose (SD) medium, grown on SD-Trp-Leu-His and SD-Trp-Leu-His-Ade. 10^–2^, diluted 100 × 10^–3^, diluted 1000 times. p53-BD/SV40-T antigen-AD was used as positive control, Lam-BD/SV40-T antigen-AD was used as negative control. **B** BIFC analysis showing that the interaction between OsWRKY62-1-cYFP,OsWRKY62-2 and OsWRKY45-nYFP forms a functional YFP in the nucleus. RFP: nuclear localization Marker RFP fluorescent signal; Merge: fused YFP with RFP fluorescent signal. All assays have been repeated at least three times with similar results. bar = 20 μm

**Fig. 6:**
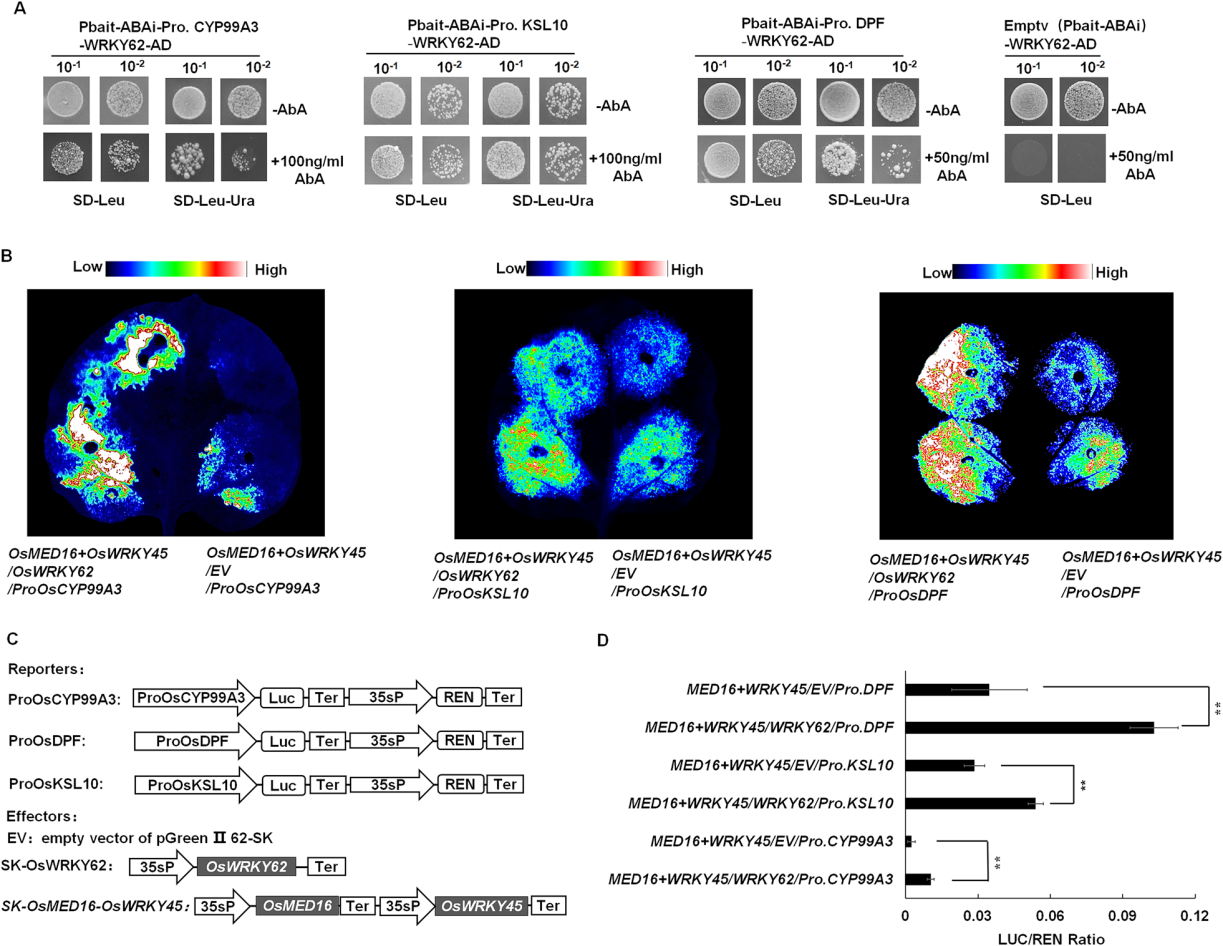
OsMED16, OsWRKY45 and OsWRKY62 co-expression can further synergistically activate diterpenoid phytoalexin biosynthesis genes transcription. **A** Y1H experiment indicating OsWRKY62 binding to *OsCYP99A3*, *OsKSL10* and *OsDPF* promoters. Negative control was performed with the empty vector pbait-ABA. Yeast cells were grown on SD-Leu and SD-Leu-Ura containing different concentrations of Aureobasidin A (AbA) at 30 °C for 3–5 d. 10^–1^, diluted 10 × 10^–2^, diluted 100 times. **B** Analysis of the effects of OsWRKY45 + OsMED16 and OsWRKY45 + OsMED16 + OsWRKY62 on *proOsCYP99A3, proOsKSL10* and *proOsDPF*. Luminescence imaging was performed in leaves of *N. benthamiana* at 80 h after co-infiltration with the indicated vectors. **C** Schematic diagram of the trans-activation assay effector and reporter constructs. The reporter vector carries the renilla luciferase gene under the control of the 35S promoter and the firefly luciferase reporter gene under the control of the *OsCYP99A3, OsKSL10,* and *OsDPF* promoters. Ter, cauliflower mosaic virus terminator; REN, Renilla luciferase; LUC, firefly luciferase; 35sP, cauliflower mosaic virus 35S promoter. **D** Luciferase activity analysis using 35S: OsMED16-OsWRKY45 and 35S: OsMED16-OsWRKY45/35S: OsWRKY62 as effectors and *ProOsCYP99A3*:Luc, *ProOsKSL10*:Luc, and *ProOsDPF*: Luc as reporters, respectively. The LUC/REN activity ratios are the means ± SD; n = 3. Comparisons were performed with Student’s t-test. **P < 0.01

The previous study has shown that OsWRKY62 interacts with OsWRKY45 to form a heterodimer that activate the expression of OsDPF (Fukushima et al. 2016). And the 1:1 yeast two hybrid was performed between OsMED16 and OsWRKY45 to confirm this interaction.The Y2H assay shows that, consistent with the previous study, OsWRKY62-1 and OsWRKY62-2 interact with OsWRKY45 (Fig. 5A). BIFC assays showed that both OsWRKY62-1 and OsWRKY62-2 interact with OsWRKY45 and these interactions occur in the nucleus (Fig. 5B). However, Neither OsWRKY62-1 nor OsWRKY62-2 interacts with OsMED16 (Fig. 5A). Further Y1H assay showed that OsWRKY62 can directly bind to the promoters of *OsCYP99A3, OsKSL10* and *OsDPF* (Fig. 6A). This indicates that OsWRKY62 may be involved in the transcriptional regulation of genes related to the biosynthesis of diterpenoid phytoalexins. To test how the OsWRKY62 protein participates in the OsMED16-OsWRKY45 complex to regulate target genes’ expression, we first employed the Y1H assay to detect whether OsWRKY62 (OsWRKY62-1) binds to the promoters of *OsCYP99A3*, *OsKSL10*, and *OsDPF*. As shown in Fig. 6A, OsWRKY62 was able to bind directly to the promoters of *OsCYP99A3*, *OsKSL10*, and *OsDPF*. This indicates that OsWRKY62 is probably involved in diterpenoid phytoalexin biosynthesis-related gene transcriptional regulation.

Next, the dual luciferase assay was employed to test how the OsWRKY62 protein participates in the OsMED16-OsWRKY45 complex to regulate the expression of target genes. Compared with 35S::OsWRKY45 and 35S::OsMED16 co-expressed. the expression levels of *proOsCYP99A3::LUC*, *proOsKSL10::LUC*, and *proDPF::LUC* found to be further activated when 35S::OsWRKY45,35S::OsMED16 and 35S::OsWRKY62 were co-expressed (Fig. 6B–D, Additional file 1: S12 and S13). As the data shown above, the OsMED16-OsWRKY45-OsWRKY62 complex was able to bind to the promoter regions of several phytoalexin synthesis-associated genes to promote the transcriptional level. Compared with the OsMED16-OsWRKY45 complex, the OsMED16-OsWRKY45-OsWRKY62 complex could further synergistically activate phytoalexin synthesis-related gene transcription.

## Discussion

### The Multiple Roles of Mediator in Plant Disease Resistance

The regulation of gene transcription in higher eukaryotes is a complex and delicate process that requires the involvement of many components including gene-specific transcription factor, RNA polymerase II, general transcription factors (GTFs), and mediator complex (MED), in which MED plays a central role in the gene transcription regulation (Kornberg 2005). Since the first report of mediator complexes in plants in 2007, abundant subsequent works have confirmed that mediator complexes in plants have significant roles in the regulation of gene transcription. With the development of bioinformatics and gene function research methods in recent years, there have been several reports on the involvement of subunits of the mediator complex in plant defense. For instance, In *Arabidopsis*, *MED25* was found to regulate plant disease resistance defense depending on the jasmonic acid (JA) signaling pathway; Subsequent studies found that MED25 interacted with MYC2, a transcription factor of the jasmonic acid pathway, revealing a clearer MED25-mediated defense pathway(Kidd et al. 2009; Cevik et al. 2012). In a similar vein, *AtMED8, 12, 13, 16, 21*, and *CDK8* have been found to regulate resistance to necrotrophic pathogens in the jasmonate/ethylene (JA/ET) depended manner (Dhawan et al. 2009; Zhang et al. 2012; Zhu et al. 2014; Zhai et al. 2019). The *AtMED16* was identified to enhance resistance to *Pseudomonas syringae* in *Arabidopsis* by controlling both SA- and JA-mediated defense gene expression (Wathugala et al. 2012). The above results illustrate that plant MEDs are involved in plant defense responses through multiple signaling pathways.

*Magnaporthe oryzae* is a hemibiotrophic fungus. It causes rice blast, the most devastating disease of food crops*.* Although plant MEDs have been researched extensively in *Arabidopsis*, the mediator complex's molecular mechanisms in crop immunity to hemibiotrophic fungus is not well clarified. In this work, we describe the role of *OsMED16,* a tail subunit of the rice MED, in positively regulating the response to rice blast (Fig. 1C–E). We demonstrated that *OsMED16* regulates plant immunity via positively regulating phytoalexin synthesis by forming a complex with OsWRKY45 and OsWRKY62 to promote the transcriptional levels of several phytoalexin synthesis-related genes such as *OsCYP99A3*, *OsKSL10* and *OsDPF* (Figs. 3, 4, 6). Our results reveal a multilayered OsMED16 protein-mediated plant defense system that further deepens our understanding of disease resistance in plant MEDs.

### OsMED16 Further Enhances the Transcriptional Activity of Transcription Factors in Promoting the Transcriptional Level

In all eukaryotes, the mediator complex has a vital role in transcriptional regulation, in which RNA Pol II is involved transcriptional initiation occurs in the gene promoter region, where the chromatin remodeling complex exposes the nucleosome, which allows the transcriptional pre-initiation complex (PIC) to assemble. The mediator complex could transmit regulatory messages to the fundamental transcriptional machinery during transcription via conformational changes, forming a surface that could be flexible enough to facilitate the PIC-Mediator transcriptional initiation supercomplex assembly (Chadick et al. 2005; Chen et al. 2021, 2022). Regulation of the signal transmission from DNA-binding TFs directly to the RNA Pol II enzyme is an important function of the MEDs. During transcription, Mediator recruits RNA polymerase II by interacting with the RNA polymerase II RPB1 subunit C-terminal structural domain (CTD), while transcription factors do not bind RNA Pol II directly, but TFs can facilitate the recruitment of RNA polymerase II, thereby completing gene transcription (Myers et al. 1998; Näär et al. 2002). Mediator interaction with TF further promotes the forming of the RNA polymerase II PIC via influencing the efficiency and velocity of the reaction in a way that enhances the transcriptional activity of the TF (Cantin et al. 2003). In plants, mediator complexes can act as transcriptional activators involved in gene regulation. For instance, In Arabidopsis, MED18 associated with the TF ABI4 (abscisic acid INSENSITIVE4) further enhances the transcriptional activity of ABI4 on ABI5 (Lai et al. 2014). The amino- and carboxy-terminal portions of TaEIL1 associate with the conserved activator interaction structural domain of TaMED25 to synergistically activate the transcription of TaERF1, thus changing the basic resistance to powdery mildew in bread wheat (Liu et al. 2016). AtCDK8, a subunit of the mediator complex, promotes the expression of PR1 and NPR1 via the recruitment of RNA Pol II to their promoter regions by associating with the transcription factors TGA, WRKY6 and WRKY8 (Chen et al. 2019).

**Fig. 7:**
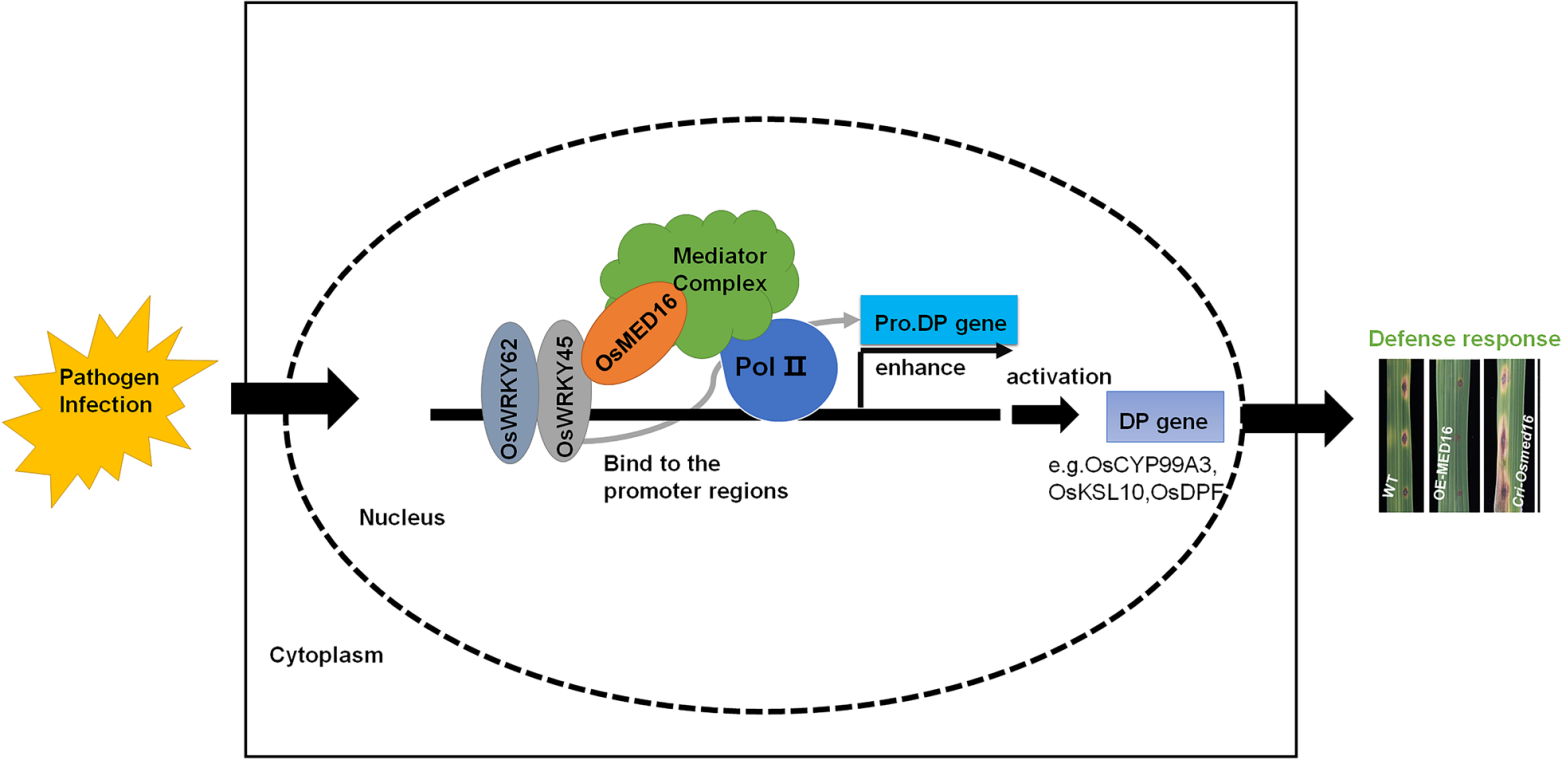
Proposed working model of the OsMED16 in the rice immune response against *Magnaporthe oryzae.* When *Magnaporthe oryzae* infection is perceived d by rice, On the one hand, OsWRKY45 will interact with OsMED16 to recruit RNA Pol II to the promoter region of downstream target genes of OsWRKY45, and activate the expression of target genes such as *OsCYP99A3*, *OsKSL10*, and *OsDPF*. On the other hand, OsWRKY62 will interact with OsWRKY45 to form a heterodimer, and then transmit the information to the Mediator complex through the interaction of OsWRKY45 and OsMED16, which will recruit more RNA Pol II to activate the expression of downstream DP genes. Finally, the immune response to *Magnaporthe oryzae* was enhanced in rice. Pol II, RNA polymerase II. DP gene, diterpenoid phytoalexin biosynthesis gene

In our research, we showed that once OsMED16 was additionally added into the system with OsWRKY45 or OsWRKY45-OsWRKY62 complex, the expression levels of *OsCYP99A3*, *OsKSL10*, and *OsDPF* were further activated (Figs. 4, 6), which indicates that when OsMED16 is present, more RNA polymerase II can be enrolled into the promoter region to enhance the transcription levels. In summary, OsMED16 acts as a central function in the regulation of plant phytoalexin genes, and OsMED16 could enhance the transcriptional activity of OsWRKY45 and OsWRKY45-OsWRKY62 heterodimers by recruiting more RNA Pol II (Fig. 7).

### Role of OsMED16 Gene in Disease Resistance of Rice

In Arabidopsis, *AtMED16* is a positive regulator of plant disease resistance defense (Wathugala et al. 2011; Zhang et al. 2012). In this study, The *OsMED16* that we cloned encodes 1170 amino acids, or named OsSRF6 of 3513 bp as reported by Wathugala et al. (Wathugala et al. 2011); 1–393 bp truncated of the 3903 bp Os10g0498700-01at 5′ end. The expression of *OsMED16* (*OsSFR6*) in the *atmed16* (*atsfr6*) mutant background restored the visible phenotype of the wild type. This suggests that there is a functional similarity between *OsMED16* (*OsSFR6*) and *AtMED16* (Wathugala et al. 2011). Our experiments showed that overexpressing *OsMED16(OsSFR6)* positively regulates rice resistance to rice blast in a *Nipponbare* background (Fig. 1C, D), which is similar to the function of *AtMED16*, However, this differs from recent studies showing that overexpression of full-length *OsMED16* (3906 bp) in “Zhonghua 11” as a negative regulator, In their study, they found that overexpression of the *OsMED16* gene (3906 bp) in japonica rice Zhonghua 11 “ZH11” enhanced rice susceptibility to rice blast, with no obvious lesion mimic phenomenon in the leaves (Zhang et al. 2023). Another functional research on *OsMED16* (3906 bp) is Jiang et al. (Jiang et al. 2021). They found that overexpression of *OsMED16* in *Nipponbare* resulted in growth inhibition, yield reduction and the appearance of lesion mimic phenotype. RNA-Seq analysis showed that overexpression of *OsMED16* resulted in the up-regulation of a large number of disease resistance-related genes, including the disease resistance gene *OsWRKY45*, which is relevant to our study, as well as the widely reported resistance genes to rice blast, *OsPR1a*, *OsPR1b* and *OsPAL1*. Maybe it was the different backgrounds used that made the Jiang et al. and Zhang et al. overexpression plant exhibits different phenotypes. We analyzed the differences between *OsMED16* (*OsSFR6* 3513 bp) and *OsMED16* (3906 bp) and found that the 131 amino acids lacking in our cloned *OsMED16* (*OsSFR6*) was enriched with Gly (41/131) which may be the reason for the differences in disease resistance functions between *OsMED16* (*OsSFR6*) and *OsMED16*(3906 bp). The reason for the opposite disease resistance regulatory functions exhibited by *OsMED16* (3513 bp) and *OsMED16* (3906 bp) could also be that using different background materials.

Interestingly, when we knocked out *OsMED16* using the CRISPR/Cas9 system, we obtained double-stranded and single-stranded effectively edited lines (Additional file 1: Fig. S1A and Fig. 1B). Consistent with previous studies, the double-stranded knockdown lines we obtained also showed seedling lethality (Additional file 1: Fig. S1B), but only the single-stranded effectively edited lines showed unrestricted growth. Further identification of disease resistance revealed that the knockout strain was more susceptible to rice blast disease than the wild type (Fig. 1C, D), which was similar to the results of Zhang et al. (2021), who silenced *OsMED16* plants using VIGS, suggesting that *OsMED16* is a positive regulator of rice blast disease in rice. The reason for this difference from the recently reported (Zhang et al. 2023) findings of *OsMED16* knockout plants being more resistant to disease could be that phenotypic differences between knockouts and knockdowns could be caused by genetic compensation responses, as in plants (Gao et al. 2015; Wang et al. 2023) and zebrafish (Ma et al. 2019). Alternatively, the observed disparity could potentially be attributed to varietal differences and variations in the pathogens employed across the studies.

### OsMED16 may also Increase Rice Resistance Against Rice Blast by Regulating H_2_O_2_ Accumulation

Hydrogen peroxide was a signal for plant disease resistance and it had a toxic effect on pathogenic (Li et al. 2021). It has been found that rice disease-resistant rice would accumulate hydrogen peroxide more readily than disease-susceptible rice (Li et al. 2014). In this study, we also found that the accumulation of H_2_O_2_ was significantly increased in the *OsMED16* overexpression plants (Additional file 1: Fig. S8A and B). Meanwhile, the expression of H_2_O_2_ synthesis-related genes was increased in *OsMED16* overexpression lines and decreased in knockout lines (Additional file 1: Fig. S8C). The expression of H_2_O_2_ degradation-related genes exhibited the opposite trend (Additional file 1: Fig. S8D).

Although we found that many H_2_O_2_ synthesis and degradation-related genes have W-boxes on their promoters (Additional file 1: Fig. S9A), However, the dual-luciferase reporter system assay results indicated that OsWRKY45 and OsMED16 did not enhance the expression of H_2_O_2_ synthesis and degradation-related genes (Additional file 1: Figs. S9B and S10). Therefore, it is hypothesized that OsMED16 regulation of hydrogen peroxide synthesis in rice is not dependent on OsWRKY45, and there may be other transcription factors recruited by OsMED16 for the regulation of H_2_O_2_ synthesis.

## Methods and Materials

### Plant Material, Growth Conditions

The *Nipponbare* variety wild type (WT) rice (*Oryza sativa* L.subsp. *Japonica*) was used as control material and transgenic analysis, the rice was grown during the normal season in experimental fields in Wuhan, China (average daily temperature about 28 °C). *Nicotiana Benthamiana* seeds were planted in nutrient soil in pots and maintained at 25 °C ambient temperature, 60% relative humidity and 8 h/16 h/dark/light photoperiod in a greenhouse*.*

### Phylogenetic Analysis

We obtained protein sequences homologous of MED16 from 10 different species from the NCBI (https://www.ncbi.nlm.nih.gov/) database by BLAST search. Multiple sequence comparison was performed using the Clustal W program. The aligned sequences were used to construct a phylogenetic tree in MEGA6 using the neighbour-joining method, with PoissionModel, partial deletion and bootstrap parameters replicated 1000 times.

### Vector Construction and Rice Transformation

The 3513 bp coding sequence of the OsMED16 gene was amplified and inserted into the pU1301-CaMV35S vector, which was linearised using Kpn I and BamH I enzymes (Additional file 1: Fig. S3). This was done using the ClonExpress II one-step cloning kit (Vazyme, Nanjing, China). The resulting overexpression vector was then used to obtain transgenic plants through the Agrobacterium-mediated method, by inducing embryonic healing tissues from NPB. For the construction of the *CRISPR/Cas9-med16* knockout vector, we designed two gRNAs based on the *OsMED16* DNA sequence as well as gene structure in CRISPR-P 2.0 (http://crispr.hzau.edu.cn/CRISPR2/) (Liu et al. 2017) (gRNA1: 5 'AATGCACGAGGGCATGATCG 3′; gRNA2: 5′ GTGTTCACATTGCCAGGAAC3′) (Additional file 1: Supplementary Fig. S4). Based on published methods (Xie et al. 2015), three primer pairs L5AD5-F/*OsMED16*-gRNA1-U3-R, *OsMED16*-gRNA1-U3-F/*OsMED16*-gRNA2-U3R, and *OsMED16*-gRNA2-U3-F/L5AD5-R amplification were performed by PCR, and three PCR products were diluted 20–50 times and mixed in equal volumes. S5AD5-F/S5AD5-R primer pairs were used for amplification, and the resulting PCR products were then cloned onto the pRGEB32 vector using the In-Fusion HD Cloning Kit. Subsequent Agrobacterium EHA105-mediated transformation of rice callus was performed to obtain *OsMED16* knockout transgenic plants. Primers used in this study were listed in Additional file 2: Supplemental Table S1.

### RNA Extraction and Expression Analysis

Rice total RNA was extracted using the RNA Extraction Kit (Promega, USA), and performed according to the instructions provided by the manufacturer. A GoScript reverse transcription kit was used to synthesise the first strand of cDNA, amplified from 1 μg of total RNA as described in the instructions and finally diluted 1 0 0 times with ddH_2_O. The expression of the relevant genes was assessed using the rice OsActin1 gene (LOC_Os03g50885) as an internal control. Realtime fluorescent quantitative PCR (RT-qPCR) was conducted utilizing SYBR Green Master Mix (Applied Biosystems, USA) on a CFX Connect Real-time System (Bio-RAD, USA) Each experiment consisted of three biological replicates and three technical replicates.

### Pathogen Preparation, Infection

The *M. oryzae* isolate *Guy11* was kindly provided by the research team of Professor Huang Junbin (Huazhong Agricultural University). *M. oryzae* preparation and inoculation were carried out in accordance with published methods (Li et al. 2017), The *M. oryzae* strain *Guy11* was inoculated on complete agar and cultivated in a climatic chamber at 25 °C for 7–9 days until the mycelium covered the entire petri dish. Subsequently, the strain was eluted into tomato oat medium with ddH_2_0, and after 4–5 days of incubation in a 25 °C climatic chamber, *Guy11* spores were eluted with 0.25% tween water, and 8–10 μl were placed on leaves in the middle of four-leafed stage plants, which were grown in a 12 h/12 h (day/night) climatic chamber at a temperature of 26 °C and relative humidity of 90% for 5 d. The experiment was repeated three times and the lesion sizes was measured using ImageJ 1.32j software. It was repeated three times for all experiments and measurement of lesion sizes on inoculated leaves using ImageJ 1.32j software. The determination of the relative infection rate of blast fungus in rice cells was carried out as previously described (Deng et al. 2017), and at least 15 DNA samples were tested for each inoculation.

### Hydrogen Peroxide Detection

The inoculated isolated leaves were stained with 0.5 mg/mL DAB staining solution, and the procedure was to place the inoculated isolated leaves in a vacuum oven with a centrifuge tube containing DAB staining solution and then evacuated for 30 min and incubated overnight at room temperature with shade.The leaves were washed with ddH_2_O and placed in a solution of acetone: methanol = 1:1 to remove the dechlorophyll. Pictures were taken with a stereo microscope (Leica, VT1000S, Germany). The detection of H_2_O_2_ was performed according to the H_2_O_2_ quantitative analysis kit (Shanghai Sangon, China).

### Yeast Two-Hybrid (Y2H) Assay

Following the manufacturer’s instructions, we used Match-maker Library Construction and Screening Kits (Clontech) to construct a rice cDNA library from leaves infected with *Guy11* for use in this investigation.In 1:1 yeast two hybrid system, for the construction of the baited vector BD-OsMED16, BD-OsMED19 and BD-OsWRKY45, full-length CDS of OsMED16, OsMED19 and OsWRKY45 were amplified with corresponding primer pairs primers and cloned into the vector pGBKT7, and subsequently transformed into yeast strain Y2H. The full-length CDS of OsMED16, OsWRKY62-1 and OsWRKY62-2 were amplified with corresponding primer pairs and cloned them into pGADT7 and subsequently transformed into yeast strain Y187. The positive plasmid-transformed Y187 and Y2H strains were selected on SD-Leu-Trp, SD-Leu-Trp-His and SD-Leu-Trp-His-Ade media to confirm the interaction, according to the experimental objectives (Hu et al. 2018).

### Bimolecular Fluorescence Complementation (BiFC) Assay

To generate the BiFC constructs, the full length of OsMED16 OsWRKY62-1, OsWRKY62-2 and OsWRKY45 were amplified with corresponding primer pairs and inserted into pS1301nYFP or pS1301cYFP vectors between BamHI and SalI (Yuan et al. 2010). The plasmids of OsMED16-cYFP, OsWRKY45-nYFP, OsMED16-cYFP, pS1301cYFP and the nuclear marker [mCherry-AtHY5, Zhao et al. (2019)] were transformed into *Agrobacterium tumefaciens* strain GV3101 by chemical transformation and subsequently co-infiltrated into leaves of *Nicotiana benthamiana* (Chen et al. 2008; Hu et al. 2021a, b). Fluorescence signals in leaf epidermal cells were observed using a confocal microscope (Olympus FV1200).

### Yeast One-Hybrid (Y1H) Assay

Yeast one-hybrid assays were performed using the Matchmaker One Hybridisation System Manual (Clontech) to confirm the interaction between OsMED16, OsWRKY45, OsWRKY62-1 and the promoters of their hypothetical targets. To obtain the bait vector, the promoter sequence was inserted into the pAbAi vector (Additional file 1: Fig. S5). The full-length of OsMED16, OsWRKY45, and OsWRKY62-1 were inserted into the pGADT7 vector to obtain OsMED16-AD, OsWRKY45-AD, OsWRKY62-1-AD as the prey vector, and the two vectors were transformed into the Y1HGold strain. Yeast cells were cultured for 3–5 d at 30 °C on SD/-Leu and SD/-Ura-Leu synthesis media supplemented with the optimal ABA (Aureobasidin A) concentration according to the screening assay for optimal concentrations to suppress bait reporter strains (Additional file 1: Fig. S6) to identify the interaction between OsMED16, OsWRKY45, OsWRKY62-1 and each promoter.

### Dual-Luciferase (LUC) Reporter Assays

The promoter sequences of *OsCYP99A3*, *OsKSL10*, *OsDPF*, *OsRbohB*, *OsRbohF*, *OsCAT*-B, *OsGPX2*, *OsAPX4*, *OsAPX5* and *OsAPX7* were amplified with *ProOsCYP99A3-0800-F/R*, *ProOsKSL10-0800-F/R*, *ProOsDPF-0800-F/R*, *ProOsRbohB-0800-F/R*, *ProOsRbohF-0800-F/R*, *ProOsCAT*-B*-0800-F/R*, *ProOsGPX2-0800-F/R*, *ProOsAPX4-0800-F/R*, *ProOsAPX5-0800-F/R* and *ProOsAPX7-0800-F/R* primer pairs, and then respectively inserted into pGreenII 0800 vector at *Hind III* and *BamH I* sites to obtain the *ProOsCYP99A3-LUC*, *ProOsCYP99A3-LUC*, *ProOsDPF-0800-LUC*, *ProOsRbohB-LUC*, *ProOsRbohF-LUC*, *ProOsCAT*-B*-LUC*, *ProOsGPX2-LUC*, *ProOsAPX4-LUC*, *ProOsAPX5-LUC* and *ProOsAPX7-LUC* vectors as reporter constructs. The full lengths of OsMED16, OSWRKY45 and OsWRKY62-1 were amplified with OsMED16-SK-F/R, OsWRKY45-SK-F/R and OsWRKY62-SK-F/R primers. The resulting PCR products were then inserted into the pGreenII 62-SK vector respectively at the *BamH I* and *ECOR I* sites to obtain the effectors SK-OsMED16, SK-OsWRKY45, and SK-OsWRKY62 vectors. and the pGreenII 62-SK vector without any DNA insertion was used as the empty vector control. To obtain SK-OsMED16-OsWRKY45 and SK-OsMED16-OsWRKY62 vectors, we first modified the pGreenII 62-SK vector as shown in the attached Additional file 1: Fig. S7. Firstly, the full-length NOS terminator was ligated into the 62-SK vector to obtain the 62-SK-NOS vector, Subsequently, after *SalI* digestion, the full length of the 35 s promoter was inserted into the 62-SK-NOS vector, and finally construct an expression vector D35s-62-SK with two 35 s promoters in tandem. Next, the full length of OsMED16 was inserted into the D35s-62-SK vector to generate the SK-35 s-MED16 vector. Subsequently, the full lengths of OsWRKY45 were inserted into the SK-35 s-MED16 vector to obtain SK-OsMED16-OsWRKY45 vectors as effectors. The luminescence imaging analysis was performed according to the previously reported method (Chen et al. 2008; Hu et al. 2021a, b). The activity analysis of LUC and REN was performed according to the method described in the instructions of the dual luciferase kit (Sangon, Shanghai, China). The ratio of Luc activity were performed using a multi-mode plate reader (PerkinElmer, USA) to calculate to control REN activity.

## Conclusion

In this study, we investigated the function of OsMED16, a subunit of the rice mediator factor complex, to gain new insights into the regulation of rice resistance to rice blast. Here, we found that OsMED16 (OsSFR6) promotes the transcript levels of several genes related to phytoalexin synthesis by forming a complex with OsWRKY45 and OsWRKY62, thereby enhancing rice resistance to rice blast.The dual mechanisms of functional differences between OsMED16/OsSFR6 (3513 bp) and OsMED16 (3906 bp) are also worthy of further investigation.

### Supplementary Information

**Additional file 1**. **Fig. S1**. The homozygous of CRISPR/cas9 edited OsMED16 plants caused seedling lethality. **Fig. S2**. Phylogenetic tree of MED16 proteins. **Fig. S3**. Mapping of the pU1301-1-CaMV35S-OsMED16-Flag overexpression vector. **Fig. S4**. Mapping of CRISPR/Cas9-*Osmed16* knockout vectors. **Fig.S5**. Diagram of Pbait-ABAi-Pro.CYP99A3, Pbait-ABAi-*ProKSL10*, Pbait-ABAi-*Pro.DPF* vector construction. **Fig. S6**. Screening assay for optimal concentrations to suppress bait reporter strains. **Fig. S7**. Diagram of carrier modification for pGreen II 62-SK. **Fig. S8**. OsMED16 positively regulates rice resistance to rice blast via modulating H_2_O_2_ biosynthesis. **Fig. S9**. The analysis and cloning of promoters of H_2_O_2_ synthesis and degradation-related genes. **Fig. S10**. The H_2_O_2_ synthesis and degradation-related genes promoter activation were not dependent on the OsMED16-OSWRKY45 pathway. **Fig. S11**. OsMED16 not interacting with *Pro.CYP99A3, Pro.KSL10, Pro.DPF*. **Fig. S12**. Analysis of the effects of OsWRKY45+OsWRKY62 and OsWRKY45+OsWRKY62+OsMED16 on *proOsCYP99A3, proOsKSL10 and proOsDPF*. **Fig. S13** Analysis of effects of Ev, OsWRKY45, OsMED16+OsWRKY45 and OsMED16+OsWRKY45/OsWRKY62 on proOsCYP99A3, proOsKSL10 and proOsDPF, respectively, detected in one leaf.

**Additional file 2**. **Table S1**. Primer used in this study.

## Data Availability

All relevant data in this study can be found within the manuscript and its supporting information files.

## Abbreviations

RNA Pol II: RNA polymerase II
DP: Diterpenoid phytoalexin
qRT-PCR: Reverse transcription quantitative PCR
WT: Wild-type
OE: Overexpression
JA: Jasmonic acid
ET: Ethylene
SA: Salicylic acid
MED: Mediator complex
GTFs: General transcription factors
PIC: Pre-initiation complex
CTD: C-terminal structural domain
TF: Transcription factor
NPB: Nipponbare
AbA: Aureobasidin A
