# Supplementary material for: The Mediator Subunit OsMED16 Interacts with the WRKY Transcription Factor OsWRKY45 to Enhance Rice Resistance Against Magnaporthe oryzae
Source: Rice (N Y). 2024 Apr 1;17:23. doi: 10.1186/s12284-024-00698-9 (PMC10984912; doi:10.1186/s12284-024-00698-9)
Supplement: Supplementary file 1 — Additional file 1. Fig. S1. The homozygous of CRISPR/cas9 edited OsMED16 plants caused seedling lethality. Fig. S2. Phylogenetic tree of MED16 proteins. Fig. S3. Mapping of the pU1301-1-CaMV35S-OsMED16-Flag overexpression vector. Fig. S4. Mapping of CRISPR/Cas9-Osmed16 knockout vectors. Fig.S5. Diagram of Pbait-ABAi-Pro.CYP99A3, Pbait-ABAi-ProKSL10, Pbait-ABAi-Pro.DPF vector construction. Fig. S6. Screening assay for optimal concentrations to suppress bait reporter strains. Fig. S7. Diagram of carrier modification for pGreen II 62-SK. Fig. S8. OsMED16 positively regulates rice resistance to rice blast via modulating H2O2 biosynthesis. Fig. S9. The analysis and cloning of promoters of H2O2 synthesis and degradation-related genes. Fig. S10. The H2O2 synthesis and degradation-related genes promoter activation were not dependent on the OsMED16-OSWRKY45 pathway. Fig. S11. OsMED16 not interacting with Pro.CYP99A3, Pro.KSL10, Pro.DPF. Fig. S12. Analysis of the effects of OsWRKY45+OsWRKY62 and OsWRKY45+OsWRKY62+OsMED16 on proOsCYP99A3, proOsKSL10 and proOsDPF. Fig. S13 Analysis of effects of Ev, OsWRKY45, OsMED16+OsWRKY45 and OsMED16+OsWRKY45/OsWRKY62 on proOsCYP99A3, proOsKSL10 and proOsDPF, respectively, detected in one leaf. [file 12284_2024_698_MOESM1_ESM.docx]

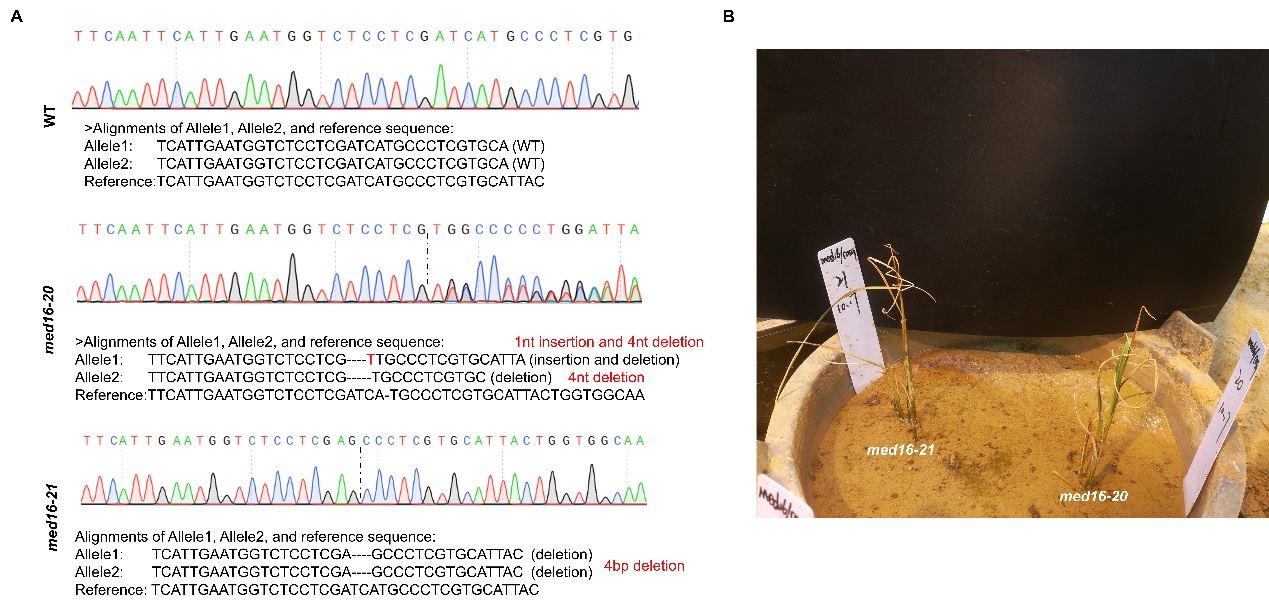


**Figure S1. The homozygous of CRISPR/cas9 edited *OsMED16* plants caused seedling lethality.** (A) Sanger sequencing results showed that compared with wild type, the single strand of the sgRNA1 region in *Osmed16* genomic DNA had a deletion of 1nt, one strand of the sgRNA1 region in *med16-20* genomic DNA had an insertion of 1nt and a deletion of 4nt, and the other strand had a deletion of 4nt, and the sgRNA1 region in *med16-21* genomic DNA had a deletion of 4bp.

(B) Phenotypes of seedlings of *med16-20* and *med16-21* lines


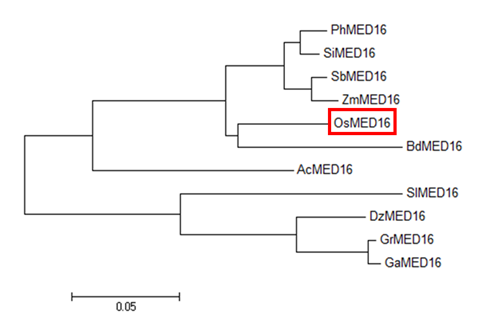


**Figure S2. Phylogenetic tree of MED16 proteins.**

Phylogenetic analysis of OsMED16 and MEDs from other plants: Panicum hallii (Ph), Setaria italica (Si), Sorghum bicolor (Sb), Zea mays (Zm), Brachypodium distachyon (Bd), Ananas comosus (Ac), Solanum lycopersicum (Sl), Durio zibethinus (Dz), Gossypium raimondii (Gr) and Gossypium arboreum (Ga). The neighbor-joining tree was constructed using the MEGA6 program.


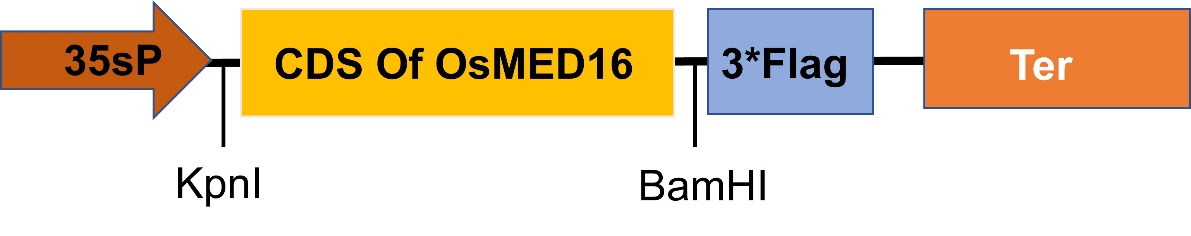


**Figure S3. Mapping of the pU1301-1-CaMV35S-OsMED16-Flag overexpression vector**

35sP, cauliflower mosaic virus 35s Promoter; Ter, nopaline synthase terminator.


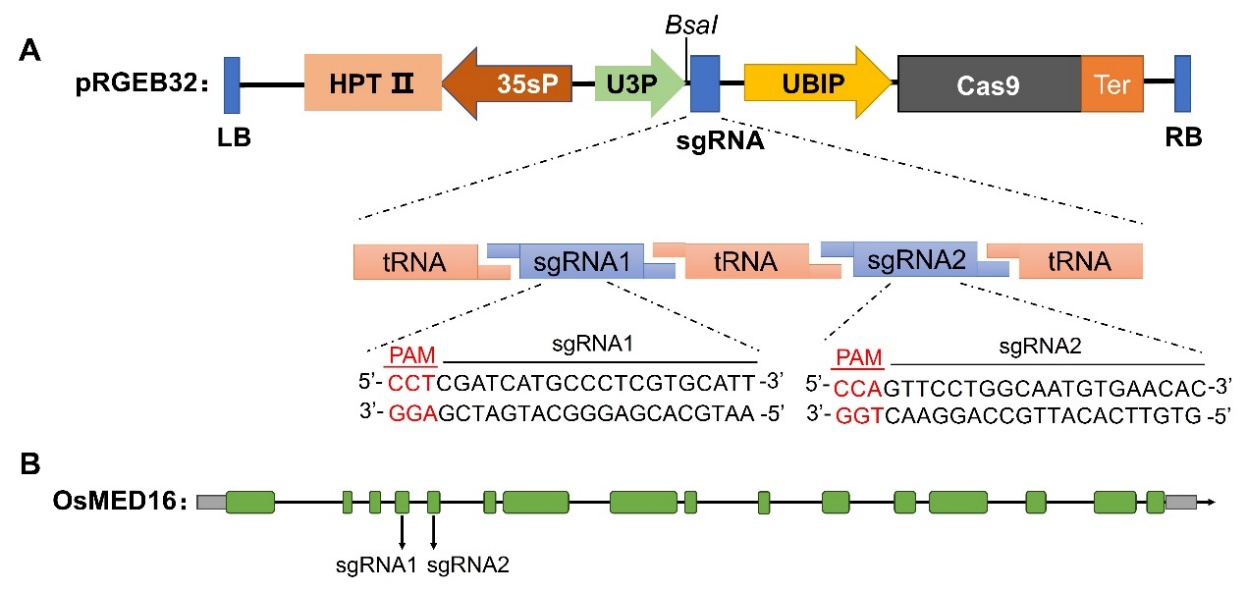


**Figure S4. Mapping of CRISPR/Cas9-*Osmed16* knockout vectors.**

(A) The pRGEB32 vector skeleton is shown on the top, the 20-base pair targeting sequence (sgRNA in black letters) is shown at the bottom, and the red letters indicate the PAM structure. 35sP, cauliflower mosaic virus 35S promoter; UBIp, rice ubiquitin promoter; U3p, rice U3 snoRNA promoter; HPT II, hygromycin phosphotransferase II; Ter, nopaline synthase terminator; LB, T-DNA left border; RB, T-DNA right border. (B) Position of sgRNA1 and sgRNA2 in the schematic representation of the OsMED16 genomic DNA.


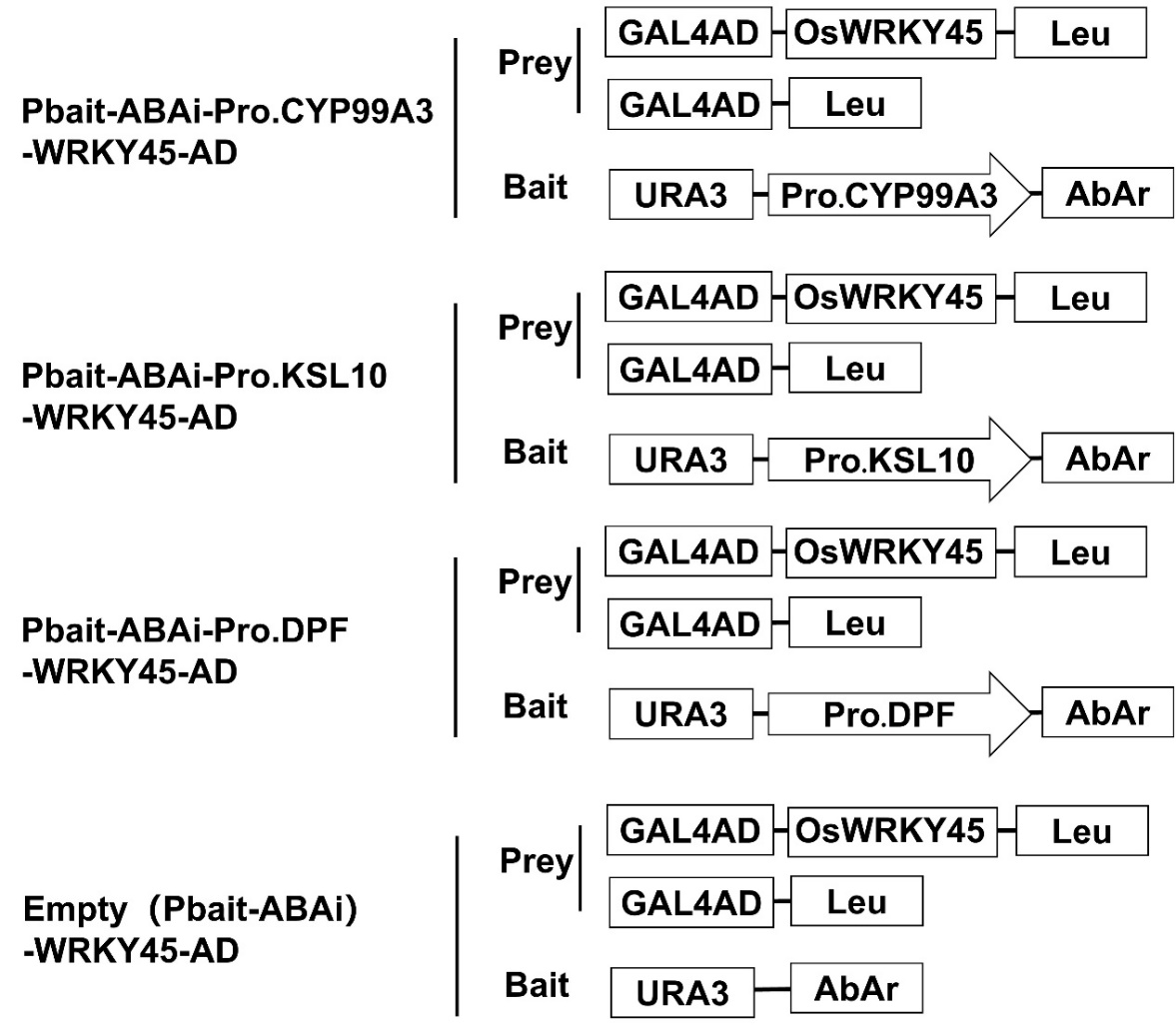


**Figure S5.** **Diagram of Pbait-ABAi-Pro.CYP99A3, Pbait-ABAi-ProKSL10, Pbait-ABAi-Pro.DPF vector construction.**


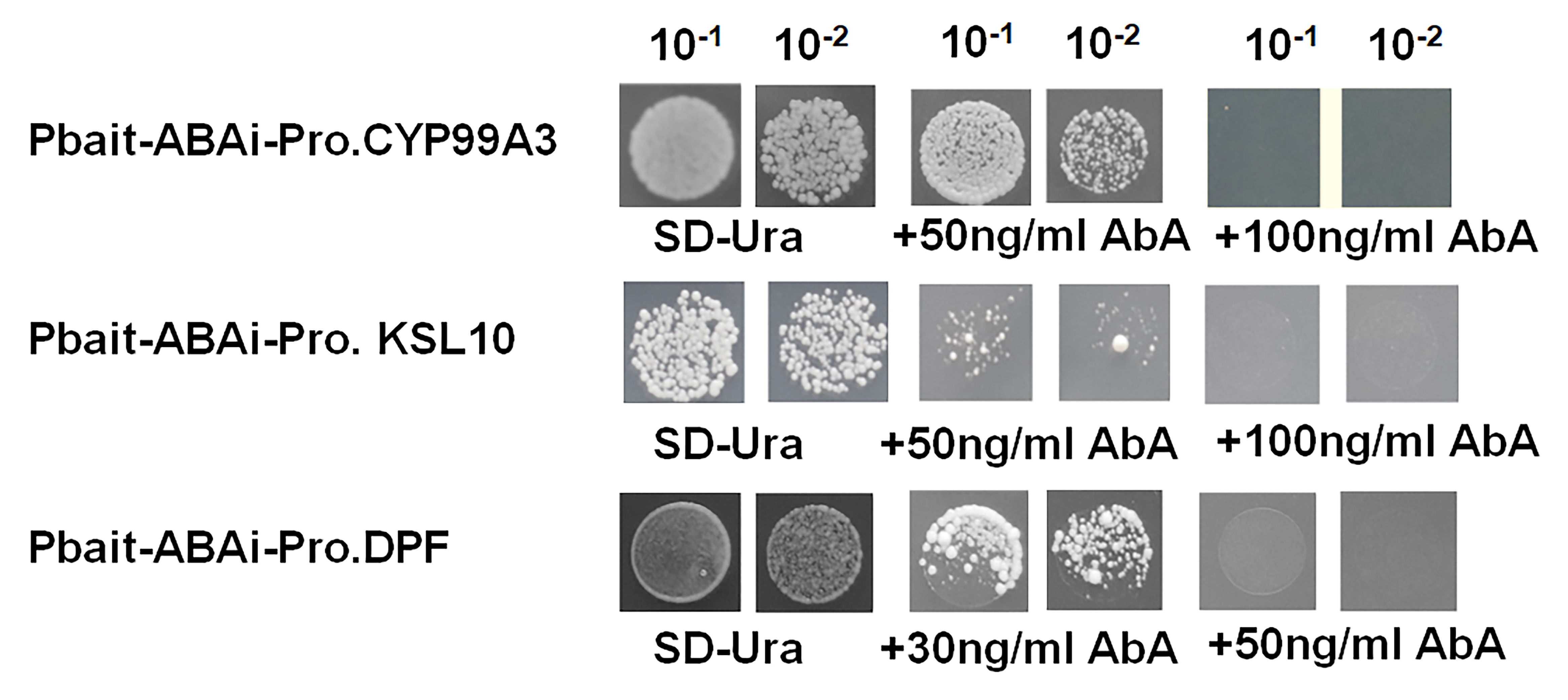


**Figure S6. Screening assay for optimal concentrations to suppress bait reporter strains.**

The bait reporter strain was grown in a medium containing SD-Ura with different concentrations of Aureobasidin A（AbA）at 30°C for 3~5d. The optimal concentration to inhibit the bait reporter strain was determined based on the growth.


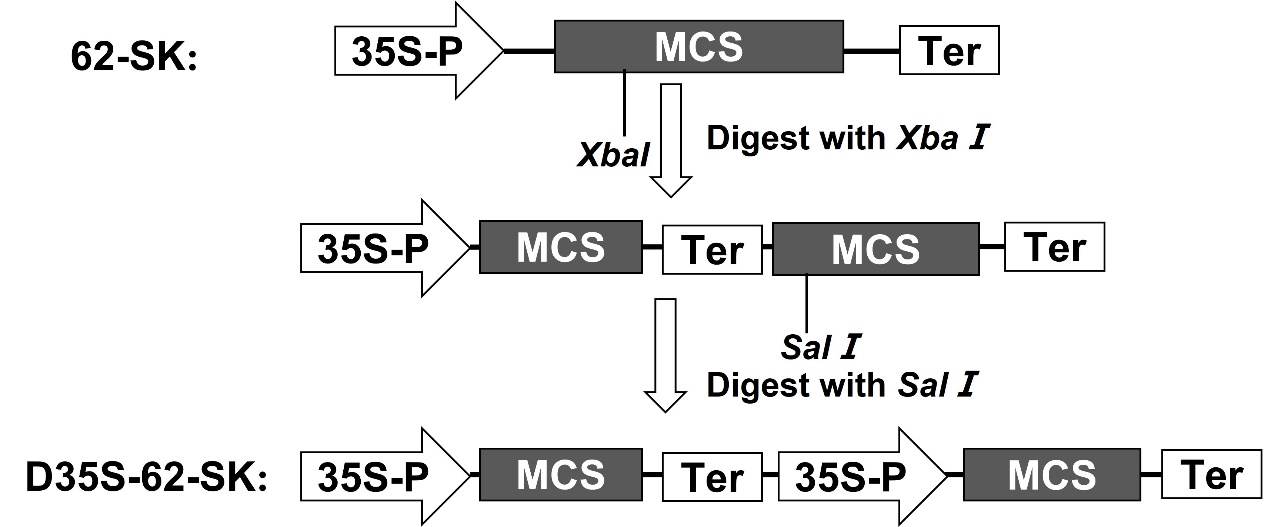


**Figure S7. Diagram of carrier modification for pGreen Ⅱ 62-SK.**

Modifying the original pGreen II 62-SK vector by inserting a NOS terminator and 35s promoter. The original vector was first digested with XbaⅠ and the full-length Nos terminator was ligated using the In-Fusion HD cloning kit (Clontech), and then the 35s promoter was ligated using the In-Fusion HD cloning kit (Clontech) after SalⅠ digestion, and finally, an expression vector was constructed which could express both proteins, named D35s-62-SK


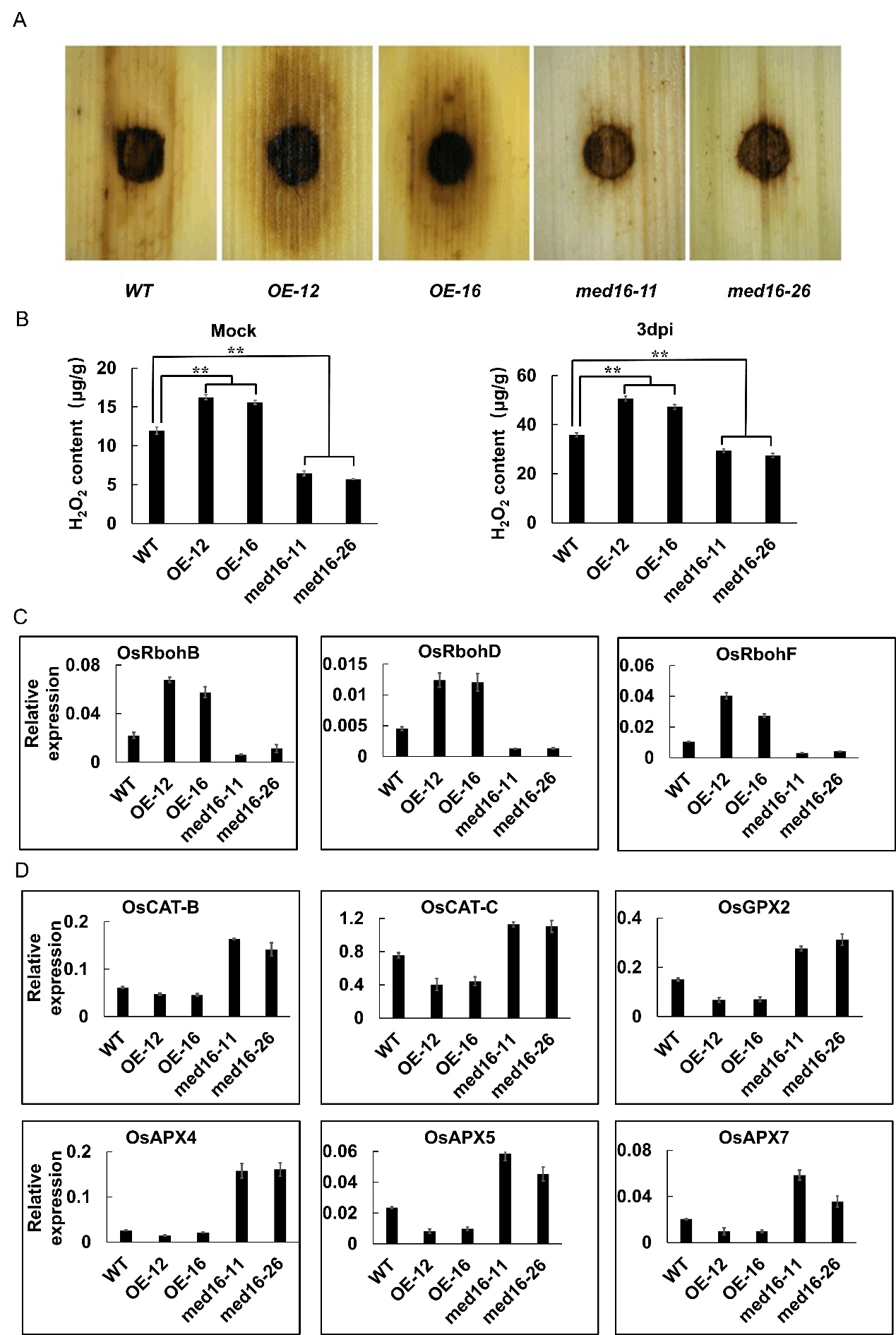


**Figure S8. *OsMED16* positively regulates rice resistance to rice blast via modulating H_2_O_2_ biosynthesis.**

(A) DAB staining observation of leaves of WT and *OsMED16* transgenic lines after 3 d infestation with *M.oryzae*. (B) H_2_O_2_ content of WT and *OsMED16* transgenic lines under normal and after 3 d infestations with *M.oryzae*（3dpi）. Values are means ± SD; n = 9. Comparisons were performed with Student’s t-test. *, P < 0.05; **, P < 0.01. (C) qRT-PCR analysis of the expression levels of H_2_O_2_ synthesis-related genes in transgenic and wild-type plants at 3 days after *M.oryzae* inoculation. The values are normalized to *OsACTIN1* and expressed as the means ± SD; n = 3. (D) qRT-PCR analysis of the expression levels of H_2_O_2_ degradation-related genes in transgenic and wild-type plants after 3 d infestation with *M.oryzae*.The values are normalized to *OsACTIN1* and expressed as the means ± SD; n = 3.


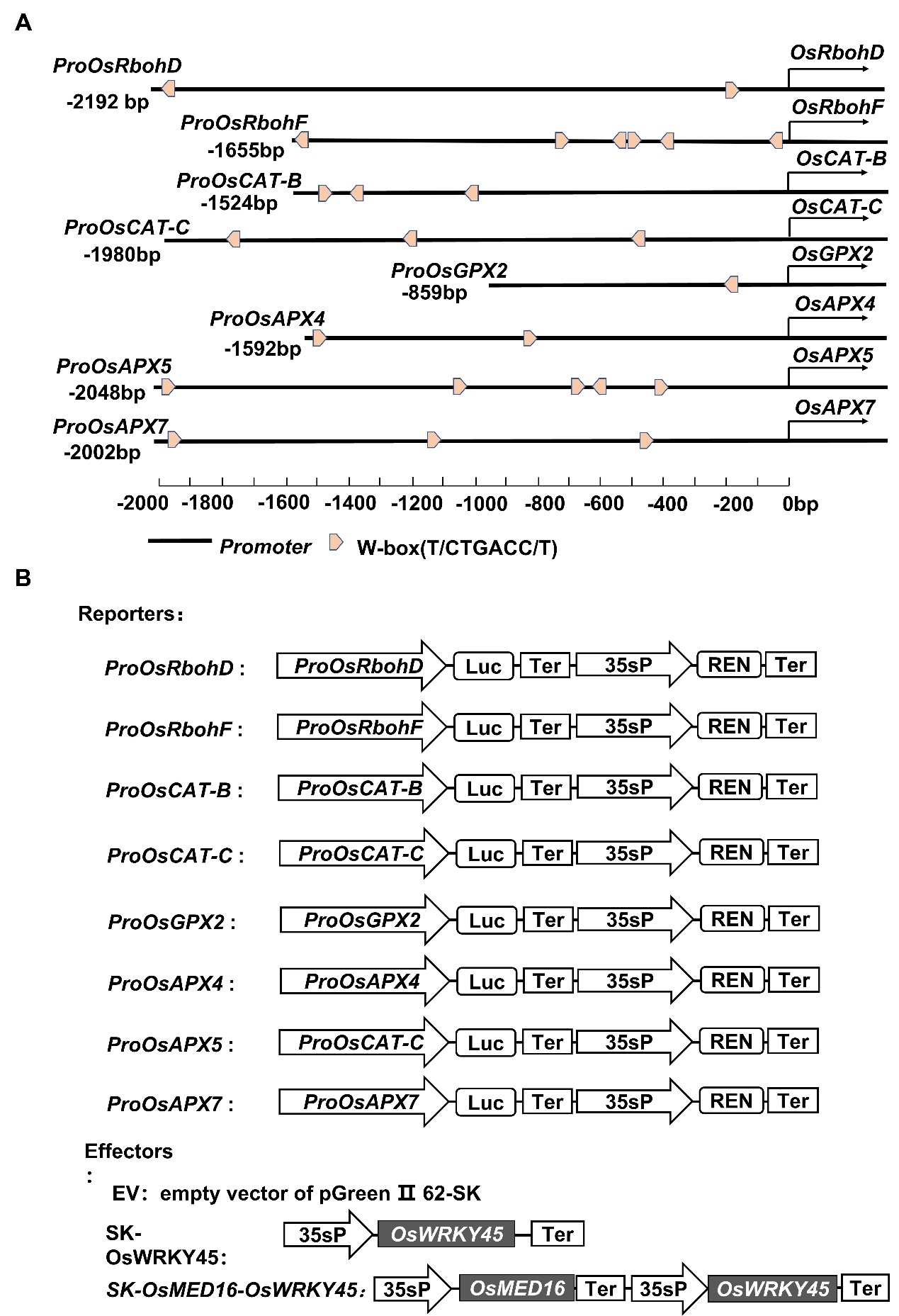


**Figure S9. The analysis and cloning of promoters of H_2_O_2_ synthesis and degradation-related genes**

(A) W box position diagram of *OsRbohD, OsRbohF, OsCAT-B, OsCAT-C, OsGPX2, OsCAT-B, OsAPX4, OsAPX5* and *OsAPX7* promoters. (B) Schematic diagram of the trans-activation assay effector and reporter constructs. The reporter vector carries the renilla luciferase gene under the control of the 35S promoter and the firefly luciferase reporter gene under the control of the *OsRbohD, OsRbohF, OsCAT-B, OsCAT-C, OsGPX2, OsCAT-B, OsAPX4, OsAPX5* and *OsAPX7* promoters. 35sP, cauliflower mosaic virus 35S promoter; REN, Renilla luciferase; Ter, cauliflower mosaic virus terminator; LUC, firefly luciferase.


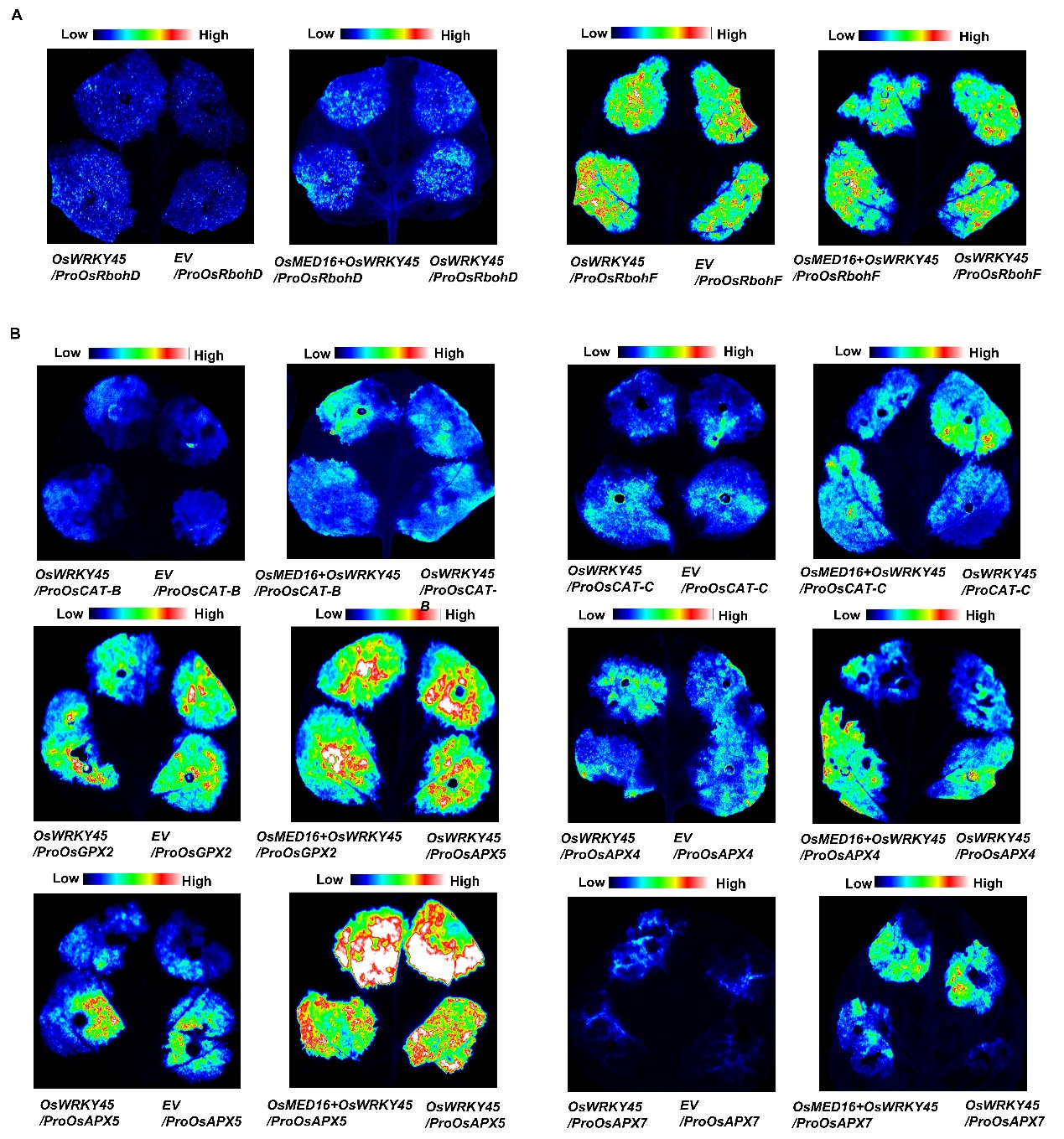


**Figure S10. The H_2_O_2_ synthesis and degradation-related genes promoter activation were not dependent on the OsMED16-OSWRKY45 pathway**

(A) Analysis of the effects of OsWRKY45 and OsWRKY45+ OsMED16 on the promoters of H_2_O_2_ synthesis-related genes (*ProOsRbohD and ProOsOsRbohF*). (B) Analysis of the effects of OsWRKY45 and OsWRKY45+ OsMED16 on the promoters of H_2_O_2_ degradation-related genes (*ProOsCAT-B, ProOsCAT-C, ProOsGPX2, ProOsAPX4, ProOsAPX5,* and *ProOsAPX7*)*.* Luminescence imaging was performed in leaves of *N. benthamiana* at 80 h after co-infiltration with the indicated vectors.


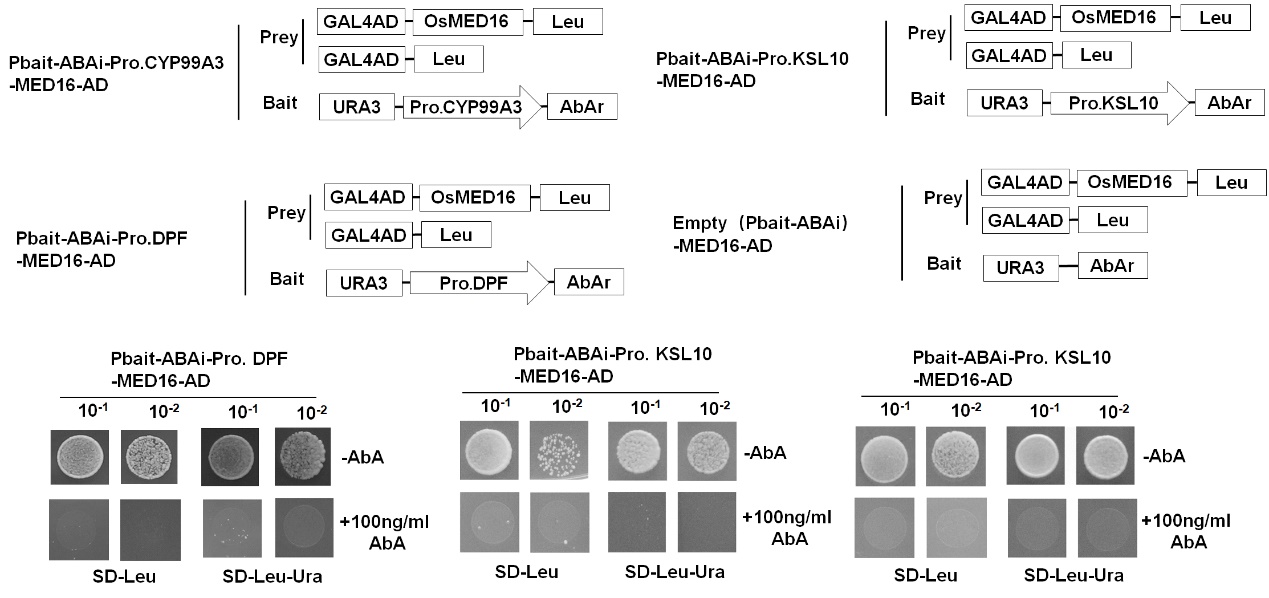


**Figure S11. MED16 not interacting with Pro.CYP99A3, Pro.KSL10, Pro.DPF.**

Y1H experiment indicating binding of OsMED16 to the promoters of OsCYP99A3, OsKSL10 and OsDPF. Yeast cells were grown on SD-Leu and SD-Leu-Ura containing different concentrations of Aureobasidin A (AbA) at 30°C for 3~5d. 10^-1^, diluted 10 times.10^-2^, diluted 100 times.


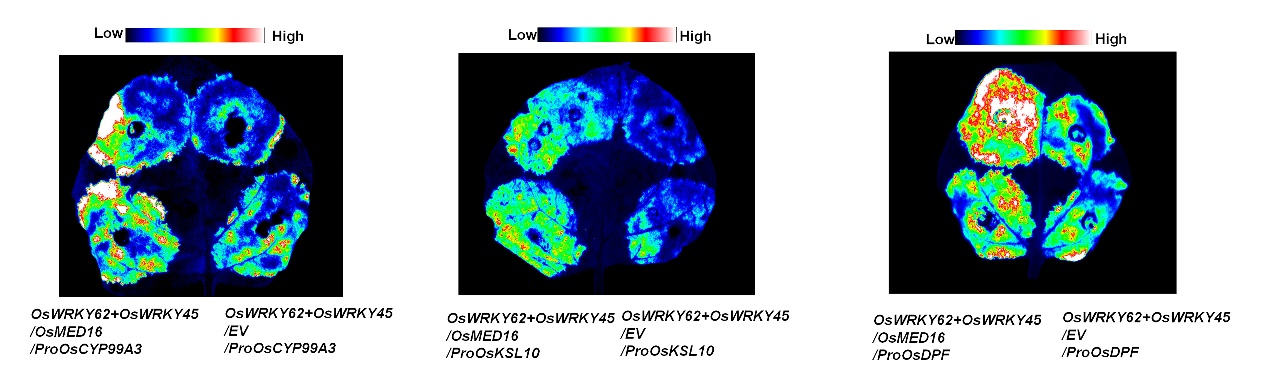


**Figure S12 Analysis of the effects of OsWRKY45+OsWRKY62 and OsWRKY45+OsWRKY62+OsMED16 on proOsCYP99A3, proOsKSL10 and proOsDPF.**

Luminescence imaging was performed in leaves of N. benthamiana at 80 h after co-infiltration with the indicated vectors.





**Figure S13 Analysis of the effects of Ev,OsWRKY45, OsMED16+OsWRKY45 and OsMED16+OsWRKY45/OsWRKY62 on proOsCYP99A3, proOsKSL10 and proOsDPF, respectively, detected in one leaf.**

Luminescence imaging was performed in leaves of *N. benthamiana* at 80 h after co-infiltration with the indicated vectors.
