# Supplementary material for: The Mediator Subunit OsMED16 Interacts with the WRKY Transcription Factor OsWRKY45 to Enhance Rice Resistance Against Magnaporthe oryzae
Source: Rice (N Y). 2024 Apr 1;17:23. doi: 10.1186/s12284-024-00698-9 (PMC10984912; doi:10.1186/s12284-024-00698-9)
Supplement: Supplementary file 2 — Additional file 2. Table S1. Primer used in this study. [file 12284_2024_698_MOESM2_ESM.docx]

**Additional file 1: Table S1**

| Gene ID （RAP-DB） | Name | Primer sequence (5ʹ-3ʹ) |
| --- | --- | --- |
| Os10t0498700-01 | OsMED16-F | ATGCGCGTGCCCGAGCTCTG |
| Os10t0498700-01 | OsMED16-R | TCAAACGACTTTCACCCATG |
|  | L5AD5-F | CAGATGATCCGTGGCAACAAAGCACCAGTGGTCTAG |
|  | L5AD5-R | TTTCTAGCTCTAAAACAAAAAAAAAAGCACCGACTCG |
|  | OsMED16-gRNA1-U3-F | AATGCACGAGGGCATGATCGgttttagagctagaa |
|  | OsMED16-gRNA1-U3-R | CGATCATGCCCTCGTGCATTtgcaccagccgggaa |
|  | OsMED16-gRNA2-U3-F | GTGTTCACATTGCCAGGAACgttttagagctagaa |
|  | OsMED16-gRNA2-U3-R | GTTCCTGGCAATGTGAACACtgcaccagccgggaa |
|  | S5AD5-F | CAGATGATCCGTGGCAACAAAGCACCAGTGGTCTAG |
|  | L5AD5-R | TTTCTAGCTCTAAAACAAAAAAAAAAGCACCGACTCG |
| Os10t0498700-01 | OsMED16-BD-F | CCATGGA GGCCGAATTCATGCGCGTGCCCGAGCTCTG |
| Os10t0498700-01 | OsMED16-BD-R | TGCAGGTC GACGGATCCTCAAACGACTTTCACCCATG |
| Os10t0498700-01 | OsMED16-AD-F | TGGAGGCCAGTGAATTCATGCGCGTGCCCGAGCTCTG |
| Os10t0498700-01 | OsMED16-AD-R | TCGAGCTCGATGGATCCTCAAACGACTTTCACCCATG |
| Os05t0322900-01 | OsWRKY45-BD-F | CCATGGAGGCCGAATTCATGAC GTCATCGATGTCGCC |
| Os05t0322900-01 | OsWRKY45-BD-R | TGCAGGTCGACGGATCCTCACAAACCCATAATGTCGTCCA |
| Os09t0417800-01 | OsWRKY62-1-AD-F | TGGAGGCCAGTGAATTCATGGACGACGACGGCGACGG |
| Os09t0417800-01 | OsWRKY62-1-AD-R | TCGAGCTCGATGGATCCCTACAAATGAACAGGAATGT |
| Os09t0417800-02 | OsWRKY62-2-AD-F | TGGAGGCCAGTGAATTCATGGAGGAGAACGCGCGGCT |
| Os09t0417800-02 | OsWRKY62-2-AD-R | TCGAGCTCGATGGATCCCTACAAATGAACAGGAATGT |
| Os03t0641700-01 | OsMED19-BD-F | CCATGGAGGCCGAATTCATGGATTCTGATGACAAGA |
| Os03t0641700-01 | OsMED19-BD-R | TGCAGGTCGACGGATCCCTAACTAAGTCCATTCCCT |
| Os10t0498700-01 | OsMED16-1301-cYFP-F | AACACGGGGGACTCTAGAATGCGCGTGCCCGAGCTCTG |
| Os10t0498700-01 | OsMED16-1301-cYFP-R | AGTACTATCGATGGATCCAACGACTTTCACCCATGTCC |
| Os05t0322900-01 | OsWRKY45-1301-nYFP-F | AACACGGGGGACTCTAGAATGACGTCATCGATGTCGCC |
| Os05t0322900-01 | OsWRKY45-1301-nYFP-R | AGTACTATCGATGGATCCAACGACTTTCACCCATGTCC |
| Os09t0417800-01 | OsWRKY62-1-1301-cYFP-F | AACACGGGGGACTCTAGAATGGACGACGACGGCGACGG |

| Gene ID （RAP-DB） | Name | Primer sequence (5ʹ-3ʹ) |
| --- | --- | --- |
| Os09t0417800-01 | OsWRKY62-1-1301-cYFP-R | AGTACTATCGATGGATCCCAAATGAACAGGAATGTGTG |
| Os09t0417800-02 | OsWRKY62-2-1301-cYFP-F | AACACGGGGGACTCTAGAATGGAGGAGAACGCGCGGCT |
| Os09t0417800-02 | OsWRKY62-2-1301-cYFP-F | AGTACTATCGATGGATCCCAAATGAACAGGAATGTGTG |
| Os03t0718100-01  Os03t0718100-01  Os10t0498700-01  Os10t0498700-01 | qOsActin-F  qOsActin-R  qOsMED16-F  qOsMED16-R | ACAGGTATTGTGTTGGACTCTGG  AGTAACCACGCTCCGTCAGG  AAATGATGTCAGACAATTGGCC  TCAACCTGTTCAAAGTTTGGAC |
| Os01t0360200-01 | qOsRbohB-F | ACAGTGCAAGTACCCACAGG |
| Os01t0360200-01 | qOsRbohB-R | CAGCAAGGCGAAGAAGAAAC |
| Os05t0465800-01 | qOsRbohD-F | CAAGTTTGTGCAGTACAGTAGG |
| Os05t0465800-01 | qOsRbohD-R | TCATCAAATGGTACGACTTTGC |
| Os08t0453700-00 | qOsRbohF-F | TGTGTAAGTAATCTGAGCCGTT |
| Os08t0453700-00 | qOsRbohF-R | AGGACGGAGATAAAAAGTAGGC |
| Os03t0358100-01 | qOsGPX2-F | GCTCGAGGATTAGATCTCGTAC |
| Os03t0358100-01 | qOsGPX2-R | CACTATTGAGCATCCCATAGGT |
| Os02t0664000-01 | qOsGPX3-F | GGAAAGTCCTCCTTATCGTCAA |
| Os02t0664000-01 | qOsGPX3-R | ACTTCTCATACAACTGGCTCAG |
| Os06t0185900-01 | qOsGPX4-F | ATGGAAAAGATGTTGCGCTTAG |
| Os06t0185900-01 | qOsGPX4-R | TTCCGTGTAATTTGCTGTTGTC |
| Os03t0285700-01 | qOsAPX1-F | TTGACAACTCTTACTTCACGGA |
| Os03t0285700-01 | qOsAPX1-R | CATCTGCAGCATATTTCTCGAC |
| Os08t0549100-01 | qOsAPX4-F | GTGAGTACTAGTCACACCACAC |
| Os08t0549100-01 | qOsAPX4-R | TTTGTGTTCACGTCATAAGTGC |
| Os12t0178200-01 | qOsAPX5-F | GTATTCTACAGAATGGGCCTGA |
| Os12t0178200-01 | qOsAPX5-R | CAGCATCAGTAGGGAGAACTAG |
| Os04t0434800-02 | qOsAPX7-F | CGTTGTGTCATCATTCCAGATG |
| Os04t0434800-02  Os02t0553200-01 | qOsAPX7-R  qOsAPX8-F | TTTGGTTTCTCGTCGAATTTGG  GCGAAATACTCCTACGGAAAGA |
| Gene ID （RAP-DB） | Name | Primer sequence (5ʹ-3ʹ) |
| Os02t0553200-01 | qOsAPX8-R | CGATCAAGAGCATGATGTTGAG |
| Os02t0115700-01  Os02t0115700-01  Os06t0727200-01  Os06t0727200-01  Os03t0131200-01  Os03t0131200-01 | qOsCAT-A-F  qOsCAT-A-R  qOsCAT-B-F  qOsCAT-B-R  qOsCAT-C-F  qOsCAT-C-R | GCCAAGCATGTGAAGAAACTAA  TCTGACATTGTCTGGCCTTATT  CATGAACTGTAATGTGTCGACC  ATACAGTTTCACAACTTGCGTC  TTAATCAGCCATGGATCCCTAC  GAGTTGTTGTTGTTCCATACGG |
| OS09T0319800-01 | qOsCPS3-F | ACAAGAAATGTCTCGAGTTCCT |
| OS09T0319800-01 | qOsCPS3-R | CTGTACAGAAACGAACTTTGCA |
| Os04t0178300-03 | qOsCPS4-F | ATCTACCCTTTGGATGTGTACG |
| Os04t0178300-03 | qOsCPS4-R | TGTCATCGATATCCTTTACCGG |
| Os04t0612000-01 | qOsKSL2-F | GAACTGTCAGATGTTCGCATTT |
| Os04t0612000-01 | qOsKSL2-R | CTTCTTTTGATCCTCCAACGTC |
| Os04t0611700-01 | qOsKSL3-F | CATCACAAAGAGGAGCTCTACT |
| Os04t0611700-01 | qOsKSL3-R | AGTTGCTGATCCTCCATTCTAC |
| Os04t0179700-01 | qOsKSL4-F | TTTGAGGTCTTTAGCGACAGAT |
| Os04t0179700-01 | qOsKSL4-R | TATAGTTGGTCCCAATGCGAAT |
| Os02t0570400-01 | qOsKSL7-F | CGAAGAATACATGGAAAGTGGC |
| Os02t0570400-01 | qOsKSL7-R | GCCTGAACAACTCATCGTATTC |
| Os12t0491800-01 | qOsKSL10-F | TATTGGACACGACATACAGGAG |
| Os12t0491800-01 | qOsKSL10-R | TCATCTGAGGTGATGTCATAGC |
| Os04t0180400-01 | qOsCYP99A2-F | ATCATGGAGATAAACTCGGCAG |
| Os04t0180400-01 | qOsCYP99A2-R | CTCTTCTTTGACGACGACTTG |
| Os04t0178400-01 | qOsCYP99A3-F | CTCAAGTTCAGCAATGGGTTC |
| Os04t0178400-01 | qOsCYP99A3-R | CCAGTATAATTGCCTTGACGTG |
| Os02t0570500-01 | qOsCYP71Z6-F | CGAAATCATAGTCGTGCTTCTG |
| Os02t0570500-01 | qOsCYP71Z6-R | GGCAAAGCAACTTGAGAATACA |
| Os02t0570700-01 | qOsCYP71Z7-F | GTCCTCCAAGACAGCACATATA |

| Gene ID （RAP-DB） | Name | Primer sequence (5ʹ-3ʹ) |
| --- | --- | --- |
| Os02t0570700-01 | qOsCYP71Z7-R | GGCAAAGCAACTTGAGAATACA |
| Os04t0179100-01 | qOsMAS-F | CAACTCGAGCTTTGGTTTCTTC |
| Os04t0179100-01 | qOsMAS-R | AAAACGTGGTTTGAAGTTGGAG |
| Os04t0179200-01  Os04t0179200-01  Os06t0569900-01  Os06t0569900-01 | qOsMAS1-F  qOsMAS1-R  qOsKOL1-F  qOsKOL1-R | CGTCAGTCGATCGACAATACTA  CAAGACAGAATCTAGCTAGCGA  GAGATTTCTTCCCCTACCTCAG  GAGATTTCTTCCCCTACCTCAG |
| Os02t0571100-01 | ProCSP2-F | CCACTTCTCCTCACCTCCTG |
| Os02t0571100-01 | ProCSP2-R | TGATACCTGGTCGCTGGATT |
| Os04t0178300-03 | ProOsCSP4-F | CCTGCCCGCCTGTGCTTGTA |
| Os04t0178300-03 | ProOsCSP4-R | GCTGGCTGCCCAAACAAGGT |
| Os02t0570500-01 | ProCYP71Z6-F | GTCGGAATCTAAACAGAAAGAAG |
| Os02t0570500-01 | ProCYP71Z6-R | GCCTTGGCTTCATGGCAGAT |
| Os02t0570700-01 | ProCYP71Z7—F | ACCTATTCGCCTCTTCCCACCTT |
| Os02t0570700-01 | ProCYP71Z7—R | GAGGACGAACAGCACTGATAGGC |
| Os04t0180400-01 | ProCYP99A2-F | GCCTAACTCACGCCAACTCC |
| Os04t0180400-01 | ProCYP99A2-R | GTCAGCAACGCCAGAAGAAT |
| Os04t0178400-01 | ProCYP99A3-F | GATTCAACATGCCACGAGAT |
| Os04t0178400-01 | ProCYP99A3-R | CCGTCCGCAGGAACATCACC |
| Os04t0179700-01 | ProKSL4-F | TTTTCTTCCCTCTTCTTCAC |
| Os04t0179700-01 | ProKSL4-R | AGGCGAAGCTGACATGAGGAC |
| Os02t0570400-01 | ProKSL7-F | CACTGCTCGCTTAGTACCGT |
| Os02t0570400-01 | ProKSL7-R | TCTGCTGCTCGGCTGCTTTG |
| Os12t0491800-01 | Pro.KSL10-F | GAGAGCTTTTACAAGAAGGCAGAAG |
| Os12t0491800-01 | Pro.KSL10-R | TCAACACCTCCTACCGCTCT |
| Os04t0179100-01 | ProMAS-F | ATCCGTTACCATTTTTCCTTCTCC |
| Os04t0179100-01 | ProMAS-R | ATCACCGCCACCTTGCCCAC |
| Os01t0196300-01 | Pro.DPF-F | GGAGCAATTAGGCTTAAAAG |

| Gene ID （RAP-DB） | Name | Primer sequence (5ʹ-3ʹ) |
| --- | --- | --- |
| Os01t0196300-01 | Pro.DPF-R | CGCACATGAAGCCGTTGTT |
| Os02t0571100-01 | Pbait-ABAi-ProCSP2-F | AAGCTTGAATTCGAGCTCCCACTTCTCCTCACCTCC |
| Os02t0571100-01 | Pbait-ABAi-ProCSP2-R | CAGAGCACATGCCTCGAGGATGGCTTCCGATCCGCG |
| Os04t0178300-03 | Pbait-ABAi-ProOsCSP4-F | AAGCTTGAATTCGAGCTCCCTGCCCGCCTGTGCTTGTA |
| Os04t0178300-03 | Pbait-ABAi-ProOsCSP4-R | CAGAGCACATGCCTCGAGGCTGGCTGCCCAAACAAGGT |
| Os02t0570500-01 | Pbait-ABAi-ProCYP71Z6—F | AAGCTTGAATTCGAGCTCGTCGGAATCTAAACAG |
| Os02t0570500-01 | Pbait-ABAi-ProCYP71Z6—R | CAGAGCACATGCCTCGAGCTTAATTGATCGTCGGCG |
| Os02t0570700-01 | Pbait-ABAi-ProCYP71Z7—F | AAGCTTGAATTCGAGCTCACCTATTCGCCTCTTCC |
| Os02t0570700-01 | Pbait-ABAi-ProCYP71Z7—R | CAGAGCACATGCCTCGAGGAGGACGAACAGCACTG |
| Os04t0180400-01 | Pbait-ABAi-ProCYP99A2-F | AAGCTTGAATTCGAGCTCGCCTAACTCACGCCAACTCC |
| Os04t0180400-01 | Pbait-ABAi-ProCYP99A2-R | CAGAGCACATGCCTCGAGAGCACGTGATATGACCTG |
| Os04t0178400-01 | Pbait-ABAi-ProCYP99A3-F | AAGCTTGAATTCGAGCTCGATTCAACATGCCACGAG |
| Os04t0178400-01 | Pbait-ABAi-ProCYP99A3-R | CAGAGCACATGCCTCGAGCGATCGATAGATAGAGGA |
| Os04t0179700-01 | Pbait-ABAi-ProKSL4-F | AAGCTTGAATTCGAGCTCTTTTCTTCCCTCTTCTTCAC |
| Os04t0179700-01 | Pbait-ABAi-ProKSL4-R | CAGAGCACATGCCTCGAGATACCAAACTGGATCAT |
| Os02t0570400-01 | Pbait-ABAi-ProKSL7-F | AAGCTTGAATTCGAGCTCTTAATTGTGAATAAAGAA |
| Os02t0570400-01 | Pbait-ABAi-ProKSL7-R | CAGAGCACATGCCTCGAGTCGCGACGATGACGACCC |
| Os12t0491800-01 | Pbait-ABAi-Pro.KSL10-F | AAGCTTGAATTCGAGCTCGAGAGCTTTTACAAGAAG |
| Os12t0491800-01 | Pbait-ABAi-Pro.KSL10-R | CAGAGCACATGCCTCGAGCGTAGTTGCCTGCACCAT |
| Os04t0179100-01 | Pbait-ABAi-ProMAS-F | AAGCTTGAATTCGAGCTCATCCGTTACCATTTTTCC |
| Os04t0179100-01 | Pbait-ABAi-ProMAS-R | CAGAGCACATGCCTCGAGGGCGCCTGAATACTAATA |
| Os01t0196300-01 | Pbait-ABAi-ProDPF-F | AAGCTTGAATTCGAGCTCGGTAGGTCGGTAGGTGAC |
| Os01t0196300-01 | Pbait-ABAi-ProDPF-R | CAGAGCACATGCCTCGAGGATCTGCTCGTTAATTAT |
|  | 35S -F | GCTTATCGATACCGTCGACTGAGACTTTTCAACAAAGG |
|  | 35S -R | GCCCCCCCTCGAGGTCGACTGTCCTCTCCAAATGAAAT |
|  | NOS-F | GGTGGCGGCCGCTCTAGAGATCGTTCAAACATTTGGCT |
|  | NOS-R | GGGATCCACTAGTTCTAGAGATCTAGTAACATAGATG |
| Os10t0498700-01 | OsMED16-SK-F | CTAGAACTAGTGGATCCATGCGCGTGCCCGAGCTCTG |

| Gene ID （RAP-DB） | Name | Primer sequence (5ʹ-3ʹ) |
| --- | --- | --- |
| Os10t0498700-01 | OsMED16-SK-R | AGCTTGATATCGAATTCTCAAACGACTTTCACCCATGT |
| Os05t0322900-01 | OsWRKY45-SK-F | CTAGAACTAGTGGATCCATGACGTCATCGATGTCGCC |
| Os05t0322900-01 | OsWRKY45-SK-R | AGCTTGATATCGAATTCTCAAAAGCTCAAACCCATAA |
| Os09t0417800-01 | OsWRKY62-SK-F | CTAGAACTAGTGGATCCATGGACGACGACGGCGACGG |
| Os09t0417800-01 | OsWRKY62-SK-R | AGCTTGATATCGAATTCCTACAAATGAACAGGAATGT |
| Os10t0498700-01 | SK-35s-MED16-F | CAGCCCAAGCTGAGCTCATGCGCGTGCCCGAGCTCTG |
| Os10t0498700-01 | SK-35s-MED16-R | CCACCGCGGTGGAGCTCTCAAACGACTTTCACCCATGT |
| Os04t0178400-01 | ProOsCYP99A3-0800-F | ACGGTATCGATAAGCTTGATTCAACATGCCACGAG |
| Os04t0178400-01 | ProOsCYP99A3-0800-R | CTAGAACTAGTGGATCCTCCTCTATCTATCGATCG |
| Os12t0491800-01 | ProOsKSL10-0800-F | ACGGTATCGATAAGCTTGAGAGCTTTTACAAGAAG |
| Os12t0491800-01 | ProOsKSL10-0800-R | CTAGAACTAGTGGATCCCGTAGTTGCCTGCACCAT |
| Os01t0196300-01 | ProOsDPF-0800-F | ACGGTATCGATAAGCTTGGTAGGTCGGTAGGTGAC |
| Os01t0196300-01 | ProOsDPF-0800-R | CTAGAACTAGTGGATCCGATCTGCTCGTTAATTAT |
| Os01t0360200-01 | ProRbohB-F | TGATTGCGGGCAACCTCC |
| Os01t0360200-01 | ProRbohB-R | AGCCCATTGGTCACCTGC |
| Os05t0465800-01 | ProRbohD-F | AGCACGAGCCGAAGCACA |
| Os05t0465800-01 | ProRbohD-R | ACGTCTTGTCGAGGAAGC |
| Os08t0453700-00 | ProRbohF-F | AGACAAGCAAAGCATCCGC |
| Os08t0453700-00 | ProRbohF-R | TCGTCGCTGTAGGCCGTGT |
| Os06t0727200-01 | ProCAT-B-F | GCTAACTTACTCCCACCG |
| Os06t0727200-01 | ProCAT-B-R | ACGCCTCTAAGCAGACATA |
| Os03t0285700-01 | ProAPX1-F | CACCTGTAGTGCGGAATC |
| Os03t0285700-01 | ProAPX1-R | GACGGGGTAGTTCTTAGCC |
| Os12t0178200-01 | ProAPX5-F | TTGGAACGGACGAATACA |
| Os12t0178200-01 | ProAPX5-R | CGAAAGAAACATCACCGATA |
| Os04t0434800-02 | ProAPX7-F | CTAAACTATGAATACGGAAGAG |
| Os04t0434800-02 | ProAPX7-R | AGAGCGGTGGTGCTGGAT |
| Os08t0453700-00 | ProGPX2-F | GTTCTTAGCGATCTAAAAGCAAAGG |

| Gene ID （RAP-DB） | Name | Primer sequence (5ʹ-3ʹ) |
| --- | --- | --- |
| Os08t0453700-00 | ProGPX2-R | TGACGACGATTAGCACCTTCC |
| Os02t0664000-01 | ProGPX3-F | TGCCAATGAACCATCCAGC |
| Os02t0664000-01 | ProGPX3-R | AGGCGCACCGACAGGGATAG |
| Os08t0549100-01 | ProAPX4-F | ATGACGGTCCCGTTCTTG |
| Os08t0549100-01  Os03t0131200-01  Os03t0131200-01 | ProAPX4-R  ProCAT-C-F  ProCAT-C-R | GATTAGGAACTAGGGGAGGGT  AACAAATGAATGCCCTCC  ATCACACCAAATTCCGCAT |
| Os01t0360200-01 | 0800-ProbohB-F | ACGGTATCGATAAGCTTTGATTGCGGGCAACCTCC |
| Os01t0360200-01 | 0800-ProRbohB-R | CTAGAACTAGTGGATCCGTTCTTCTCTACTTGTCC |
| Os05t0465800-01 | 0800-ProRbohD-F | ACGGTATCGATAAGCTTAGCACGAGCCGAAGCACA |
| Os05t0465800-01 | 0800-ProRbohD-R | CTAGAACTAGTGGATCCCCTGATCCGAACTAGGTAGC |
| Os08t0453700-00 | 0800-ProRbohF-F | ACGGTATCGATAAGCTTAGACAAGCAAAGCATCCGC |
| Os08t0453700-00 | 0800-ProRbohF-R | CTAGAACTAGTGGATCCGTCTCTCTGCGGCCGCCGC |
| Os06t0727200-01 | 0800-ProCATB-F | ACGGTATCGATAAGCTTGGGCTAACTTACTCCCACC |
| Os06t0727200-01 | 0800-ProCATB-R | CTAGAACTAGTGGATCCGGCGTGATTTGTTGTGGGA |
| Os03t0285700-01 | 0800-ProAPX1-F | ACGGTATCGATAAGCTTCACCTGTAGTGCGGAATC |
| Os03t0285700-01 | 0800-ProAPX1-R | CTAGAACTAGTGGATCCGGCTGCGGGTGGAGCTC |
| Os12t0178200-01 | 0800-ProAPX5-F | ACGGTATCGATAAGCTTTTGGAACGGACGAATACA |
| Os12t0178200-01 | 0800-ProAPX5-R | CTAGAACTAGTGGATCCGTCGGCGCCGGAGTCGAG |
| Os04t0434800-02 | 0800-ProAPX7-F | ACGGTATCGATAAGCTTAAACTATGAATACGGAAGAG |
| Os04t0434800-02 | 0800-ProAPX7-R | CTAGAACTAGTGGATCCGGGTGACGCTGGTGGTGGGT |
| Os08t0453700-00 | 0800-ProGPX2-F | ACGGTATCGATAAGCTTAAACTATGAATACGGAAGAG |
| Os08t0453700-00 | 0800-ProGPX2-R | CTAGAACTAGTGGATCCGGGTGACGCTGGTGGTGGGT |
| Os02t0664000-01 | 0800-ProGPX3-F | ACGGTATCGATAAGCTTTGCCAATGAACCATCCAGC |
| Os02t0664000-01 | 0800-ProGPX3-R | CTAGAACTAGTGGATCCCGTGGATGGGGGGCCTTCG |
| Os08t0549100-01 | 0800-ProAPX4-F | ACGGTATCGATAAGCTTATGACGGTCCCGTTCTTG |
| Os08t0549100-01 | 0800-ProAPX4-R | CTAGAACTAGTGGATCCGGCGTGGCGTCGCGTCGC |
| Os03t0131200-01 | 0800-ProCATC-F | ACGGTATCGATAAGCTTAACAAATGAATGCCCTCC |
| Gene ID （RAP-DB） | Name | Primer sequence (5ʹ-3ʹ) |
| Os03t0131200-01 | 0800-ProCATC-R | CTAGAACTAGTGGATCCGGCTGATTAATTAACGAC |
|  | MgPot2-F | ACGACCCGTCTTTACTTATTTGG |
|  | MgPot2-R | AAGTAGCGTTGGTTTTGTTGGAT |
| Os03t0234200-01 | OsUbq-F | GACGGACGCACCCTGGCTGACTAC |
| Os03t0234200-01 | OsUbq-R | TGCCAATTACCATATACCACGAC |
